# Assessing Functional Conservation Amongst *FT*- and *TFL1*-like Genes in Globe Artichoke

**DOI:** 10.3390/plants14091364

**Published:** 2025-04-30

**Authors:** Rick Berentsen, María José Domenech, Peter Visser, Francisco Madueño, Vicente Balanzà, Reyes Benlloch

**Affiliations:** 1 Instituto de Biología Molecular y Celular de Plantas, Consejo Superior de Investigaciones Científicas, Universitat Politècnica de València, 46022 Valencia, Spain; rick.berentsen@basf.com (R.B.); mdomene@ibmcp.upv.es (M.J.D.); madueno@ibmcp.upv.es (F.M.); vbalanza@ibmcp.upv.es (V.B.); 2 BASF|Nunhems, Nunhems Spain SAU, 30593 Cartagena, Spain; peter.visser@basf.com; 3 Departamento de Biología Vegetal, Facultad de Farmacia y Ciencias de la Alimentación, Universitat de València, 46100 Burjassot, Spain

**Keywords:** globe artichoke, flowering, bolting time, PEBP family, *FT*, *TFL1*

## Abstract

Globe artichoke [*Cynara cardunculus* var. *scolymus* (L.)] is a perennial composite cultivated for its immature inflorescences. Over time, the market for growers has steadily shifted away from vegetatively propagated varieties and towards seed-propagated hybrids. Since the latter tend to produce relatively late in the season, advancing the moment of flowering remains a major objective for breeders, who can benefit from insight gained into the genetic architecture of this trait. In plants, the timing of flowering is strongly regulated at the genetic level to ensure reproductive success. Genetic studies in model and non-model species have identified gene families playing crucial roles in flowering time control. One of these is the phosphatidylethanolamine-binding protein (PEBP) family, a conserved group of genes that, in plants, not only regulate the vegetative-to-reproductive phase transition, but also the development of inflorescences. In this work, we identified seven PEBP family members in the globe artichoke genome, belonging to three major clades: *MOTHER OF FT AND TFL1 (MFT)*-like, *TERMINAL FLOWER 1 (TFL1)*-like, and *FLOWERING LOCUS T (FT)*-like. Our results further show that *CcFT* expression is upregulated after the floral transition and partially complements the *ft-10* mutant, whilst *CcTFL1* is expressed in the shoot apex and developing inflorescences and complements the *tfl1-1* mutant. These results suggest that the flowering-suppressing function of *CcTFL1* is conserved in globe artichoke whereas conservation of the floral promoting function of *CcFT* remains uncertain.

## 1. Introduction

Globe artichoke [*Cynara cardunculus* var. *scolymus* (L.) Fiori], is a predominantly allogamous, perennial diploid (2n = 2x = 34) with a genome size of 1084 Mb [1]. It is mostly cultivated for immature inflorescences, called “capitulae” or more colloquially “heads”, which are valued as a vegetable. While domestication is thought to originate in the Mediterranean basin, nowadays, about 65% of its global production comes from the five highest producing countries: Italy, Egypt, Spain, Peru, and Argentina. Global production in 2020 amounted to 1516 ktonnes, increasing from 1452 ktonnes in 2016, and more than double the 708 ktonnes produced in 1961 [2].

Although the process of domestication of globe artichoke is not fully clear, it possibly started in southern Italy, from where the crop might have been spread around the Mediterranean basin by the Arabs, who dominated parts of this area around the early Middle Ages [3,4]. During this process, artichokes were selected for different traits, in particular capitulum size and shape, time of production, and yield, suggesting a relatively small set of founders and genetic base [5].

Growers traditionally treated the crop as a perennial and maintained it by vegetative propagation to ensure uniformity and conserve favorable genetics. This cultivation method is challenged by the more recent introduction of seed-propagated hybrids, which was initially driven by the elevated costs and phytosanitary issues associated with vegetative production systems [6,7]. Over time, the availability and incorporation of male sterility into breeding programs enabled the introduction of hybrid varieties that offer equal or higher yield and quality than traditional cultivars [6,8]. A challenge with commercial hybrids is that some popular seed-propagated varieties tend to produce late [9]. A possible mitigation strategy is the foliar application of gibberellic acid (GA_3_), which advances the moment of bolting if applied in a skillful manner to avoid failure and loss of quality [10,11,12].

As our recent results indicate, earliness in globe artichoke production seems linked to a reduced requirement for vernalization [13]. This supports the idea that progress in earliness can be made under a classical breeding scheme [14], and that the vernalization requirement could serve as a proxy for earliness. Given that the crop can produce one generation per year, breeding and trait introgression into elite pools are time-consuming. Modern molecular breeding approaches have the potential to speed up these processes, provided that insight is gained into the genetic architecture of earliness and flowering.

The life cycle of plants is characterized by a succession of phases, each of which needs to be precisely timed in accordance with both endogenous and environmental cues to achieve reproductive success and hence ensure the species’ fitness and survival [15]. Different gene families are known to be involved in flowering [16,17], with the phosphatidylethanolamine-binding protein (PEBP) family playing key roles as regulators of floral transition and plant architecture [18,19]. Initially isolated from bovine brain samples, the name of the PEBP gene family is derived from the capacity of the protein to interact with phosphatidylethanolamine in vitro [20,21]. Members of the family were soon discovered in plants, the first being *CENTRORADIALIS (CEN)*, from a determinate mutant of Antirrhinum majus [22]. This was followed by *TERMINAL FLOWER 1 (TFL1)* in Arabidopsis [23] and *FLOWERING LOCUS T (FT)* [24]. *TFL1* and *FT* homologs have been extensively studied in different plants, initially for their roles in the control of flowering time, inflorescence development, and architecture, and later for their role in other processes such as bud sets in tree species or seed size [18,25,26,27,28,29].

Based on sequence identity, the PEBP family in angiosperms is divided into three major clades, being *MOTHER OF FT AND TFL1 (MFT)*-like, *TERMINAL FLOWER 1 (TFL1)*-like, and *FLOWERING LOCUS T (FT)*-like. The *MFT*-like clade is considered the most ancestral and relatively little is still known about its function [30]. Different roles have been proposed, such as sporophyte development [31], determination of flowering time [32], and regulation of seed germination [23,33,34]. *MFT*-like clade members have been identified in basal lineages like mosses as well as in gymno- and angiosperms [23,30,35,36,37]. In angiosperms the ancestral *MFT* underwent a duplication event resulting in two lineages, *MFT1* and *MFT2*, which subsequently have been partially lost in different monocot and dicot clades [30,31,38].

Genes pertaining to the *TFL1*-like clade are found in spermatophytes, and they are involved in the regulation of flowering time, bud dormancy, and meristem identity, amongst others [36]. In angiosperms, the *TFL1*-like group is made up of three types of genes: *TERMINAL FLOWER 1 (TFL1)*, *CENTRORADIALIS (ATC)*, and *BROTHER OF FT AND TFL1 (BFT)*. *BFT* and *ATC* act redundantly by competing with *FT* for binding to the cofactor *FLOWERING LOCUS D (FD)* to repress floral meristem identity genes such as *APETALA1 (AP1)* [39,40,41,42,43]. In Arabidopsis, *TFL1* has long been known as both a key regulator of flowering time and of inflorescence meristem (IM) identity maintenance, which is required for indeterminate growth [23,44]. Loss-of-function mutants flower earlier than wild-type plants and form determinate inflorescences whose coflorescences are replaced by solitary flowers [45]. Expression of *TFL1* increases in the inflorescence meristem with the vegetative-to-reproductive phase change. At this stage, *TFL1* is responsible for maintaining IM identity by repressing the expression of the floral identity genes *LEAFY (LFY)*, *APETALA1 (AP1)*, and *CAULIFLOWER (CAL)*, confining the action of the latter to the auxiliary primordia [44,46]. Homologs of *TFL1* have been identified and characterized in many taxa, both in gymnosperms and in angiosperms including Asteraceae [47,48,49]. Much less information is available on the function of *ATC* and *BFT* genes. *ATC* codes for a floral repressor that is preferentially expressed under short-day conditions [39]. *BFT* overexpression in Arabidopsis resulted in a late flowering phenotype, similar to that of *35Spro::TFL1*, whereas *BFT* loss-of-function did not cause a visible phenotype in wild-type plants, although it did modify the architecture of inflorescences in the *tfl1* background. This suggests that *BFT* functions as a flowering suppressor [50]. *BFT* has also been shown to be involved in responses to salt stress [51], inflorescence architecture [50], and in bud dormancy in kiwifruit [52,53].

As opposed to *TFL1*-likes, *FT*-like genes are exclusive to angiosperms. Even if *TFL1*-like and *FT*-like genes share high sequence similarity, their roles are antagonistic. *FT* was originally identified as the florigen for its flowering promoting function and later shown to be conserved across many species [54,55]. In Arabidopsis, *FT* is a floral integrator that is expressed in the leaves upon being activated by *CONSTANS (CO)* and encodes a mobile protein that travels to the shoot apical meristem (SAM), where it controls the vegetative-to-reproductive transition by positively regulating *AP1* and *SUPPRESSOR OF OVEREXPRESION OF CONSTANS 1 (SOC1)* [56]. The flowering promoting capacity of *FT* was linked to a critical residue, Tyr85, and a single Tyr85His substitution was able to convert *FT* into a floral suppressor. Likewise, a His88Tyr substitution in the *TFL1* sequence turned the resulting protein into an *FT*-like floral activator [57]. Subsequently, Ahn et al. [58] made use of *FT*/*TFL1* chimeric proteins to discover two critical segments on exon 4 that were found to be variable in *TFL1* but highly invariable in *FT*. These two critical segments comprise a 14 a.a. B-segment, that encodes an external loop of the FT protein, and an adjacent C-segment containing a LYN/IYN triad that contacts this loop. Moreover, four more critical residues were found in exon 4 by [59] and mutations in each of the Glu-109, Trp-138, Gln-140, and Asn-152 residues could turn the FT protein into a TFL1-like floral repressor. Natural mutations exist that convert paralogs of *FT* into suppressors, such as is the case in sugar beet (*Beta vulgaris*). In this species, flowering was found to be controlled by two antagonistically acting *FT* paralogs, *BvFT1* and *BvFT2*, where the repressing effect of *BvFT1* could be attributed to three mutations in the B-segment [60]. Another *FT*-like gene, *TWIN SISTER OF FT (TSF)*, is a close homolog of *FT* and acts redundantly in the signal integration pathway involved in controlling the vegetative-to-reproductive transition [61]. Homologs of *FT* have been identified and characterized in many plant species, including in Asteraceae [62,63].

In this work, we explored the globe artichoke genome and identified seven homologs from the PEBP family. Amongst those, we functionally characterized *FT* and *TFL1* homologous genes with the objective of developing molecular markers for the floral transition in globe artichoke. We found that *CcTFL1* was able to fully complement the Arabidopsis *tfl1-1* mutant whilst *CcFT* only partially complemented the *ft-10* mutant. Moreover, *CcTFL1* was expressed predominantly in shoot apices after the vegetative-to-reproductive phase transition, whilst *CcFT* expression was limited to leaves and inflorescence structures after this transition. This suggests that *CcTFL1* is a proper functional homolog of *AtTFL1* from Arabidopsis, whilst the functional homology to Arabidopsis could not be established with certainty for *CcFT*.

## 2. Results

### 2.1. Identification of Globe Artichoke PEBP Homologs

To identify homologs of the PEBP family in globe artichoke, protein sequences of PEBP family members from Arabidopsis were used as queries in a BLASTp search against the protein sequences of globe artichoke from the Hi-C v2 genome dataset [64]. This resulted in seven significant hits, all annotated as members of the PEBP family (annotation edit distance (AED) ≤ 0.10). A protein domain search was performed and revealed significant PEBP Pfam domains in all seven proteins, further confirming their identity as PEBP family members (Supplementary Table S1).

**Figure 1:**
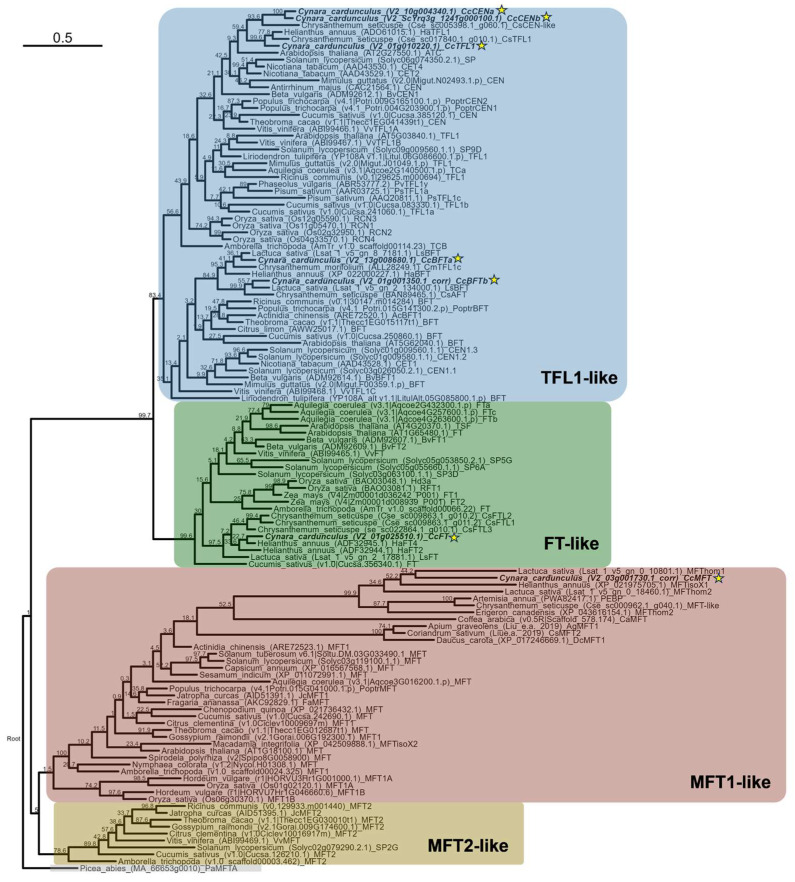
ML phylogram of PEBP genes in plants. Colors accentuate major PEBP clades, with *MFT*-likes subdivided into *MFT1*-likes and *MFT2*-likes for clarity. Gray entry is the outgroup used to root the tree. PEBP members from globe artichoke in bold and marked with a star. Node labels denote 1000-bootstrap values.

To further classify the seven globe artichoke PEBP homologs, we performed a phylogenetic study in which phylogenetic trees were constructed by clustering the globe artichoke protein sequences with those from a wide range of viridiplantae. In the phylograms (Figure 1 for maximum likelihood (ML), Supplementary Figure S1 for maximum parsimony (MP) and neighbor joining (NJ)), four major PEBP clades are apparent. These are *MOTHER OF FT AND TFL1 (MFT)*-likes, split into *MFT1*-likes and *MFT2*-likes, *FLOWERING LOCUS T (FT)*-likes, and *TERMINAL FLOWER 1 (TFL1)*-likes. In the NJ phylogram, MFT1 proteins from the campanulids (Asteraceae and Apiaceae) form a separate clade.

The split between *MFT1*-likes and *MFT2*-likes is supported by both ML, MP, and NJ, although 1000-bootstrap values were low in the ML tree. Cophenetic distances within the *MFT1*-likes are largely in line with taxonomy and evolution, albeit with some contradictions, such as the placement of the monocots and basal eudicots *Aquilegia coerulea* (blue columbine, Ranunculales) and *Macadamia integrifolia* (macadamia, Proteales). Moreover, the placement of *CaMFT* (*Coffea arabica* (coffee)), Gentianales order of the lamiids clade) and *MFT1* from *Actinidia chinensis* (kiwifruit, Ericales order) is not fully in line with taxonomy. The *MFT1*-likes are represented by one globe artichoke homolog, V2_03g001730.1, which is hence referred to as “*CcMFT*”. No homologs of *MFT2*-likes were identified in globe artichoke. *CcMFT* has a sequence similarity of 60.3% to its closest homolog *LsMFThom1* from *Lactuca sativa* (lettuce) and 41.6% to *AtMFT* from Arabidopsis.

Interestingly, *MFT1*-likes from the euasterid orders of Asterales and Apiales (campanulids) are characterized by high cophenetic distances separating the taxa, as well as the two orders, and therefore appear as a subcluster within the *MFT1*-likes, or a separate cluster in the NJ phylogram. This cluster is represented by the Asteraceae globe artichoke, lettuce, *Helianthus annuus* (sunflower), *Chrysanthemum seticuspe*, *Artemisia annua* (sweet wormwood), and *Erigeron canadensis* (horseweed), as well as by the lamiids *Coriandrum sativum* (cilantro), *Apium graveolens* (celery), and *Daucus carota* (carrot). The MFT1 protein from *Coffea arabica* (Gentianales) appears to be part of this cluster as well, although being taxonomically more distant to the former two orders. The high cophenetic distances within this subcluster suggest that the *MFT1*-like clade of *MFT* genes might be subjected to a higher rate of evolution in the campanulid families Asteraceae and Apiaceae as compared to the remainder angiosperms.

Concerning the *TFL1*-like clade, the split between *TFL1*-likes and remainder clades is well supported by NJ, MP, and ML phylograms. Moreover, these support a further subdivision of the *TFL1*-likes into two subclusters, one with proteins more similar to CEN/TFL1, and one more similar to BFT. In the ML phylogram (Figure 1), the *CEN*/*TFL1* subcluster is largely in line with taxonomy, and most nodes are well supported by 1000-bootstrap values. Three globe artichoke genes are present: V2_01g010220.1, V2_10g004340.1, and V2_ScYrq3g_1241g000100.1. On one hand, V2_01g010220.1 shares high amino acid sequence similarity with *HaTFL1* from sunflower (90.8%), *CsTFL1* from *Chrysanthemum seticuspe* (87.9%), and *AtTFL1* from Arabidopsis (68.7%). Based on these similarities, we designated V2_01g010220.1 as “*Cynara cardunculus TFL1*” (“*CcTFL1*”). On the other hand, V2_10g004340.1 and V2_ScYrq3g_1241g000100.1 are most related to *CsCEN*-like (both with 76.6% similarity), and are 98.7% similar to each other. Therefore, we named these genes *CcCENa* and *CcCENb*.

The *BFT* subcluster is predominantly composed of *BFT* homologs, with some remaining “*TFL1/CEN*” annotations suggestive of *BFT* identity as well. In fact, *CEN* orthologs *CEN1.1*, *CEN1.2*, and *CEN1.3*, from *Solanum lycopersicum* (tomato) are annotated as *BFT* orthologues on the ITAG5.0 reference genome [65]. Moreover, *BFT* identities have been suggested for *CET1* from *Nicotiana tabacum* (tobacco) and *VvTFL1c* from *Vitis vinifera* (grapevine), based on a phylogenetic study by Carmona et al., 2007 [66]. Similarly, data from [67] suggest that the antiflorigen *CsAFT* from *Chrysanthemum seticuspe* is a *BFT* homolog. For *CmTFL1c* from *Chrysanthemum morifolium*, reported as a *TFL1/CEN* homolog by [48], our results suggest it actually is a homolog of *BFT*. These considerations further strengthen the notion of this being a true *BFT* subcluster. Two *BFT* homologs were identified for globe artichoke, V2_13g008680.1 and V2_01g001350.1, which were designated as “*CcBFTa*” and “*CcBFT1b*”. Their similarities to Arabidopsis *AtBFT* are 51.1% and 72.4%, respectively.

Regarding the *FT*-like clade, we found only one globe artichoke homolog, V2_01g025510.1, which was named “*CcFT*”. It is equidistant to *HaFT4* and *LsFT*, 95.4% identical to both, whilst its similarity to *AtFT* is 74.3%. The presence of one single *FT* homolog in artichoke is in line with a report on the Asteraceae lettuce [63], while different from other Asteraceae such as sunflower and chrysanthemum, which are known to possess multiple *FT* homologs [68,69].

### 2.2. Protein Structure and Conserved Motifs in Globe Artichoke PEBP Family

With respect to gene structure, PEBP family members in most species contain four exons although exceptions have been described, like, for example, for carrots [70]. In globe artichoke, four exons were annotated in available *CcFT*, *CcTFL1*, and *CcBFTa* gene models (Supplementary Table S1). In the closely related genes *CcCENa* and *CcCENb*, the fourth exon was split into two smaller exons, effectively rendering them genes with five exons. The original *CcBFTb* gene model was missing the second exon. We reconstructed and reannotated this gene model, resulting in the four-exon model V2_01g001350.1 being corrected to V2_01g001350.1_corr. The *CcMFT* gene model was also missing the fourth exon that was not found even after manual inspection of the downstream sequences. However, we manually removed 26 bp before the start codon of exon 1 and extended the start of exon 3 by 22 bp to include the correct start codon, correcting the gene model V2_03g001730.1 to V2_03g001730.1_corr (Supplementary Table S1 and Supplementary Figure S2). The two corrected sequences were used for further analyses and, retrospectively, to correct the phylogenetic study.

**Figure 2:**
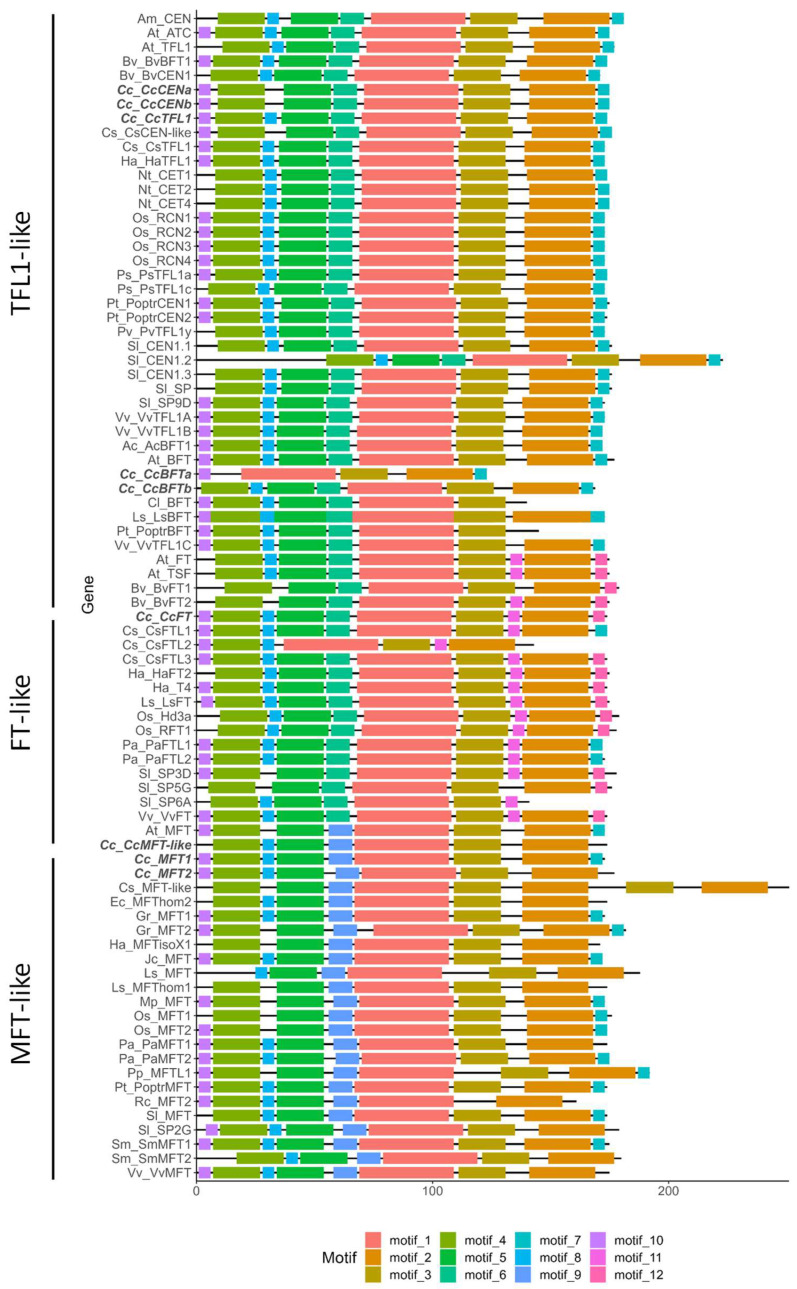
Results from MeMe motif search in PEBPs from different plant species. MeMe motif plot. Genes from *C. cardunculus* in bold. Species: Aa = *Artemisia annua*, Ac = *Actinidia chinensis*, Al = *Arctium lappa*, Am = *Antirrhinum majus*, At = *Arabidopsis thaliana*, Bv = *Beta vulgaris*, Cc = *Cynara cardunculus*, Cc = *Citrus clementina*, Cl = *Citrus limon*, Cs = *Chrysanthemum seticuspe*, Ec = *Erigeron canadensis*, Gr = *Gossypium raimondii*, Ha = *Helianthus annuus*, Jc = *Jatropha curcas*, Ls = *Lactuca sativa*, Mp = *Marchantia polymorpha*, Nt = *Nicotiana tabacum*, Os = *Oryza sativa*, Pa = *Picea abies*, Pp = *Physcomitrium patens*, Ps = *Pisum sativum*, Pt = *Populus trichocarpa*, Pv = *Phaseolus vulgaris*, Rc = *Ricinus communis*, Sl = *Solanum lycopersicum*, Sm = *Selaginella moellendorffii*, Vv = *Vitis vinifera*.

We scanned globe artichoke sequences, and other available PEBP sequences from different taxonomic groups, for conserved protein motifs to reveal putative signature sequences characteristic of particular clades. Our analysis revealed a total of 12 significant motifs (*p* < 0.05) (Figure 2, Supplementary Figure S3). *MFT*-like genes shared a conserved motif (motif 9) which was present as well in *CcMFT*. Motifs 11 and 12 were unique to the *FT*-like clade and occurred in all *FT*-like genes, except for motif 11, which was absent from SlSP5G, and motif 12, which was absent from *CsFTL1*, *PaFTL1,* and *PaFTL2*. In the latter three proteins, motif 7 was substituted for motif 12. This supports *CcFT* being a true member of the *FT*-like clade. *TFL1*-like proteins did not possess unique motifs, but the combination of the presence of motif 6 and absence of motif 10 was unique to them, with the exceptions of *CsFTL1*, *PaFTL1*, and *PaFTL2* due to the aforementioned substitution of motif 12. Motif 8 was missing in *CcCENa* and *CcCENb*, with their motif pattern being identical to that of *CsCEN*-like. Concerning the *BFT*-like genes, *CcBFTa* misses motif 4 through to motif 7, which indicates that the gene has undergone a restructuring event. *CcBFTb* motif 10 was absent, and its motif pattern was identical to *LsBFT*, supporting the result from the phylogenetic study showing that the two are similar in both sequence and structure.

### 2.3. Sequence Variation in PEBP Homologs

**Figure 3:**
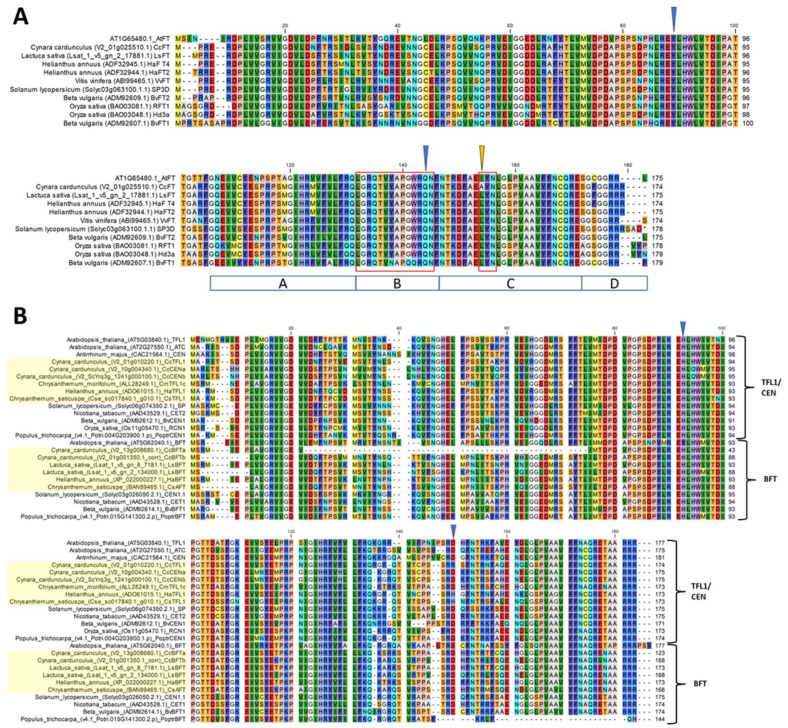
Alignment and models of *FT*-like proteins. (**A**) Protein sequence alignment of 11 selected *FT*-like proteins. Blue arrows = globe artichoke *CcFT* Tyr84 and Gln139, respectively; yellow arrow = first amino acid of LYN triad; red frame = the B-loop and LYN triad, respectively; blue boxes under the alignment = exon4 segments. (**B**) Protein sequence alignment for *TFL1*-likes. Blue arrows denote His88 and Asp144 residues.

To further study the PEBP genes from globe artichoke at the sequence level, multiple protein sequence alignments were generated for *FT*-like (Figure 3A), *TFL1*-like (Figure 3B), and *MFT*-like proteins (Supplementary Figure S4A).

The flowering promoting effect of *FT* has been attributed to the presence of critical residues in both the second exon and in the external loop coded by the fourth exon [57,58]. In particular, Tyr85 (Tyr84 in *CcFT*) and Gln140 (Gln139 in *CcFT*) are characteristic of FT and are present in proteins encoded by *CcFT* whilst being absent from *CcTFL1*, *CcCENa*, and *CcCENb* (Figure 3A). In addition to these residues, the fourth exon of *FT* harbors two conserved regions that together are required for proper *FT* function [58]. These are the canonical 14 amino acid sequence “LGRQTVYAPGWRQN” in segment B and the “LYN/IYN” triad on segment C, both encoding external loops of the protein [58]. Although the former segment was found to be fully conserved between Arabidopsis and globe artichoke, the latter showed a Leu149Ala/Ile149Ala (hydrophobic to hydrophobic) amino acid substitution, thus reading “AYN” (Figure 3A, yellow arrow). The substitution is present in both globe artichoke and cultivated cardoon, (*Cynara cardunculus* var. *altilis*) genotype ‘A41’, based on proteins predicted from whole-genome resequencing data by Acquadro et al., 2017 [71]. Moreover, the mutation appears to be unique for the genus *Cynara* and did not occur in the remainder of the *FT* references used in this study. Three-dimensional modeling of CcFT and AtFT did, however, not show a noticeable change in the structure of the external loop of the protein (Supplementary Figure S4B,C) suggesting this to be a neutral mutation.

*TFL1* is characterized by the presence of critical His88 and Asp144 residues, of which only the former is present in *CcTFL1* (Figure 3). Both residues are present in *CcCENa* and *CcCENb*. *CcTFL1* contained a unique Asp141Arg substitution at the equivalent position, whereas *HaTFL1* and *CsTFL1* possessed a Asp140His substitution at that position. This indicates that the Asp144 residue critical for TFL1 function in Arabidopsis is not fully conserved in the Asteraceae. With respect to *BFT* homologs, the critical Tyr85 and Glu141 residues [50] were found in neither *CcBFTa* nor in *CcBFTb*, and neither were they present in the remainder of the Asteraceae BFT protein sequences.

*MFT*-likes maintained the conserved D-P-D-x-P and G-x-H-R motifs, apart from *RcMFT2* from *Ricinus communis* (castor bean), the latter most likely due to a deletion causing ambiguity in the alignment. A proline residue at position 168 of *AtMFT*, reported to be unique to *MFT*-likes [30], was encountered in only 10 out of 21 sequences. The critical proline residue was absent from all Asterids, as well as from the Rosids *Citris clementina* and *Vitis vinifera*, the lycophyte *Selaginella moellendorffii* and from the liverwort *Marchantia polymorpha*. In angiosperms, the *MFT*-like group can be divided into two subclades, being *MFT1*, which have a Gly at position 111 (Supplementary Figure S3) and *MFT2*, where a Glu substitutes for it [30]. The Asteraceae *MFT*-likes studied all had a Gly at this position, indicating that they derive from the *MFT1* clade. Moreover, *MFT*-likes from the Asteraceae showed a high degree of protein sequence variation, containing 10 residues that appeared to be unique for this family.

### 2.4. PEBP Gene Expression Profiles During Globe Artichoke Development

To further characterize the PEBP genes identified in globe artichoke, we studied the expression profiles of *CcFT*, *CcTFL1*, *CcBFTa*, *CcBFTb*, and *CcCENa* in different plant tissues and the expression dynamics along the vegetative-to-reproductive phase transition. The tissues in which expression was studied were shoot apex, leaves, stem, petioles, inflorescence, root, seeds hypocotyl, and cotyledons (further detailed in Supplementary Table S4).

**Figure 4:**
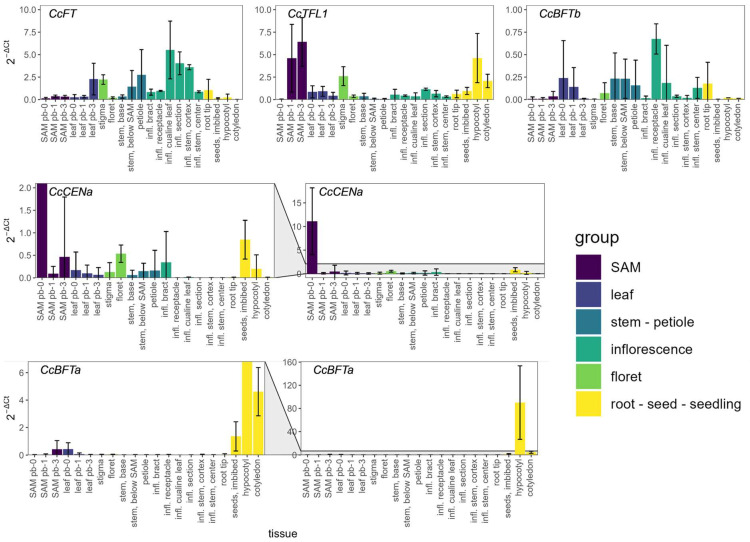
Relative gene expression of globe artichoke PEBP genes. Horizontal axis = tissue, “SAM pb-x” = shoot apex in pre-bolting stage x, “leaf pb-x” = youngest mature leaf from a plant in pre-bolting stage x, “infl.” = inflorescence. *CcCENa* and *CcBFTa* are shown both on their original scale as well as zoomed in to reveal the remainder tissues with lower relative expression values. Tissues are grouped in accordance with the legend. Error bars denote standard deviations over two biological replicates with three technical replications each. The scale of Y-axes is different between genes.

Our results show that *CcFT*, *CcTFL1*, and *CcBFTa* are expressed at higher levels than *CcCENa* and *CcBFTb* (Figure 4). *CcFT* is highly expressed in cauline leaves, stem, petioles, and inflorescence tissues. Interestingly, *CcFT* expression remains low in the shoot apex and leaves but increases in the leaves at pre-bolting stage 3 (stages according to Berentsen et al., 2024 [13]). Therefore, we observed that a *CcFT* expression increase in leaves accompanies the onset of the reproductive stage. As opposed to *CcFT*, the expression of *CcTFL1* is high in the shoot apex as early as pre-bolting stage 1. It is also strongly expressed in the stigma, the hypocotyl, and the cotyledon. We observed that *CcTFL1* expression increases in the shoot apex, along with the vegetative-to-reproductive phase transition, when inflorescence structures are formed. The *CcTFL1* expression pattern suggests that this gene has multiple functions during artichoke development, both in seedling and inflorescence development.

Expression of *CcCENa* is very high at the shoot apex at pre-bolting stage 0 and decreases steeply as the inflorescence develops, coinciding with the (end of the) vegetative phase. Furthermore, *CcCENa* expression is also detected, although weakly, in imbibed seeds and in the floret.

Expression of *CcBFTa* reaches high levels in imbibed seeds, in the hypocotyl, and cotyledons, suggesting a role during germination and/or during early seedling development. On the other hand, *CcBFTb* is expressed in the receptacle of the inflorescence, whilst expression in the remainder tissues shows high variation between replicates.

### 2.5. Assessment of Functional Conservation of CcFT and CcTFL1

Given the absence of an efficient transformation protocol for globe artichoke, we approached the functional characterization of *CcFT* and *CcTFL1* by heterologous expression in Arabidopsis. In order to test if *CcFT* functions as a flowering promoter, we cloned *CcFT* under the control of the constitutive 35S promoter and the resulting construct was used to transform plants from both the wildtype Col-0 and *ft-10* mutant. In parallel, we generated an equivalent construct to overexpress *AtFT* from Arabidopsis to compare the phenotypes. We characterized the flowering time in these transgenic plants as compared to Col-0 plants by scoring both the days until bolting and the rosette and cauline leaves produced.

**Figure 5:**
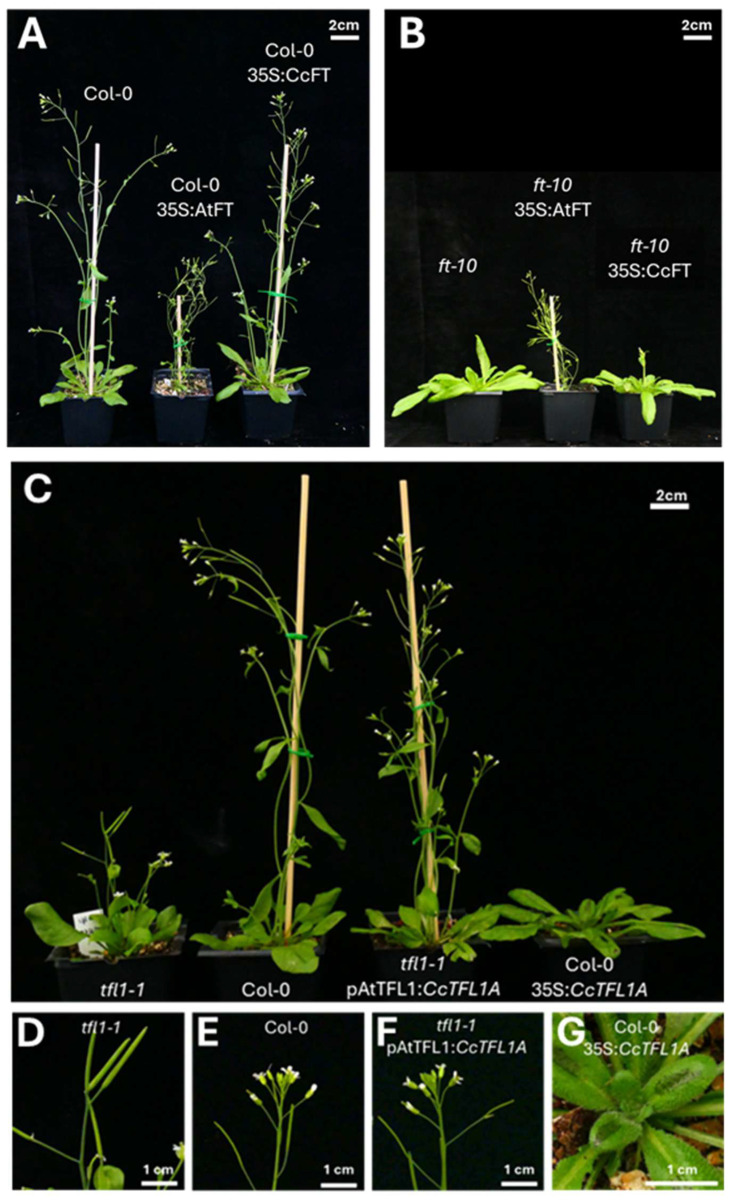
Phenotypes of *CcFT* and *CcTFL1* expressed in Col, *ft-10*, and *tlf1-1* mutants. (**A**) Overexpression of *CcFT* and *AtFT* in *ft-10* background (30 days after sowing (das)). (**B**) Overexpression of *CcFT* and *AtFT* in Col-0 background (30 das). (**C**) Gross aspect of *CcTFL1* expressed in *tfl1-1* mutant and Col-0 under both the native *TFL1* promoter and a 35S overexpression promoter (30 das). (**D**) Close-up of *tfl1-1* inflorescence (30 das). (**E**) Close-up of Col-0 inflorescence (30 das). (**F**) Close-up of pAtTFL1:*CcTFL1A tfl1-1* (30 das). (**G**) Close-up of center of rosette of 35S:*CcTFL1A* Col-0 (30 das).

**Figure 6:**
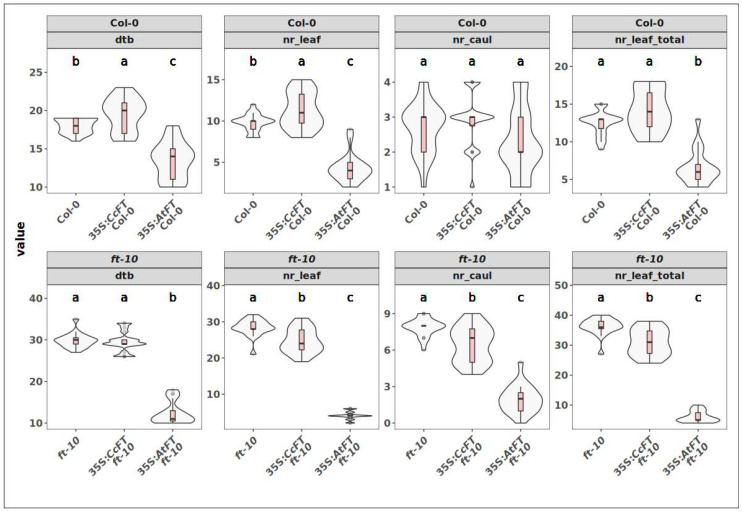
Observations of phenotypic effects of overexpression of *CcFT* in Col-0 and *ft-10* mutant. Top row: effects of overexpression of *CcFT* and *AtFT* in the wild type Col-0 background. Bottom row: effects of overexpression of *CcFT* and *AtFT* in the ft-10 mutant background. Phenotypes: “dtb” = days to bolting, “nr_leaf” = maximal number of rosette leaves before bolting, “nr_caul” = number of cauline leaves on the inflorescence, “nr_leaf_total” = total number of leaves. Boxplots inside the violin plots: lines = highest/lowest 1.5× interquartile range, red boxes = lower/upper quartiles, middle line = median. Compact letter displays summarize results of pairwise comparisons between genotypes.

Overexpression of the two aforementioned constructs in Col-0 resulted in two T_1_ populations of 20 individuals each, 35S:*CcFT* Col-0 and 35S:*AtFT* Col-0, which were compared to 20 Col-0 control plants. Notably, 35S:*AtFT* T_1_ individuals appeared weaker and produced smaller rosettes than those from Col-0 whereas this was not the case for 35S:*CcFT* individuals (Figure 5A). With respect to flowering, under our growth conditions, Col-0 plants bolted between 16 and 19 days after sowing (das). Compared to that, 60% of the 35S:*CcFT* Col-0 population bolted later than the latest-bolting Col-0 plant, and remarkably, no plants bolted earlier than the earliest Col-0 plant. Bolting in the 35S:*CcFT* Col-0 population occurred in the range of 20–23 das, with a mode of 20 das. Contrarily, 85% of the 35S:*AtFT* Col-0 population bolted earlier than the earliest Col-0 plants, whilst no plant bolted later than the latest-bolting Col-0 plants. Bolting in the 35S:*AtFT* Col-0 population occurred between 10 and 15 das, with the mode being 14 das (Figure 6, Supplementary Tables S5 and S6). This indicates that heterologous overexpression of *CcFT* did not cause a flowering-promoting effect. On the contrary, *CcFT* had a significantly delaying effect on flowering, since the number of rosette leaves in 35S:*CcFT* Col-0 plants was higher as compared to Col-0 plants (35% showed more leaves than the maximum observed in any of the Col-0 plants). This was, again, not the case amongst the 35S:*AtFT* Col-0 population, in which 90% of the plants had lower numbers of rosette leaves than Col-0 plants (Figure 6). With respect to the number of cauline leaves, we did not observe any transgenics with values outside of the range of Col-0. When we analyzed the total leaf number, comprising rosette and cauline leaves, we observed a similar number as described for rosette leaves. These data indicate that, as opposed to *AtFT* overexpression, *CcFT* overexpression did not cause a reduction in leave numbers, and therefore it does not show a flowering-promoting effect when overexpressed in a heterologous system (Figure 6).

Overexpression of 35S:*CcFT* and 35S:*AtFT* constructs in the *ft-10* mutant resulted in 19 35S:*CcFT ft-10* and 20 35S:*AtFT ft-10* T_1_ plants. These were compared to 20 *ft-10* controls. The phenotype of *ft-10* is late-bolting and only 17% of the 35S:*CcFT ft-10* plants bolted slightly earlier than the earliest-bolting *ft-10* plants, whilst none bolted later than *ft-10*. Contrarily, 35S:*AtFT ft-10* plants bolted within a range of 10–18 das, which is significantly earlier than *ft-10* plants, which start bolting around 27 das (Figure 6). When these results are compared to Col-0 (16–19 das), none of the 35S:*CcFT ft-10* individuals bolted within that range, indicating that *CcFT* was not capable of complementing the *ft-10* mutant, even when overexpressed. This is in contrast to 35S:*AtFT ft-10* plants, of which 79% bolted even earlier than Col-0 (Figure 5B and Figure 6), suggesting a strong flowering-promoting effect. Numbers of rosette leaves in 35S:*CtFT ft-10* were slightly, though significantly, lower than those in *ft-10* at 30 das. In 35S:*AtFT ft-10* transgenics, however, rosette leaf numbers were considerably smaller (Figure 5B). When the number of rosette leaves is considered, 6% of 35S:*CcFT ft-10* plants (one single individual) had less leaves than *ft-10*, whereas all 35S:*AtFT ft-10* plants had less rosette leaves than *ft-10*, and even less than Col-0. The number of cauline leaves in 39% of the 35S:*CcFT ft-10* plants was lower than the minimum observed in *ft-10* (4–5 leaves vs. the minimum of 6 cauline leaves for *ft-10*). This contrasts with 35S:*AtFT ft-10*, for which all plants had lower numbers than *ft-10* (0–5 cauline leaves). When we analyzed the total leaf number, comprising rosette and cauline leaves, we observed a similar number as described for rosette leaves. These results indicate that *CcFT*, in contrast to *AtFT*, does not fully complement the *ft-10* mutant with regard to the number of rosette and cauline leaves.

We generated two constructs for heterologous expression of *CcTFL1*: One under the control of a 35S constitutive promoter and another using an Arabidopsis, native, *TFL1* (*AtTFL1*)-promoter. We transformed these constructs in wild-type Col-0 plants and *tfl1-1* mutant plants. This resulted in a total of 64 T_1_ plants, comprising 26 35S:*CcTFL1* Col-0, 9 35S:*CcTFL1 tfl1-1*, and 29 pAtTFL1:*CcTFL1 tfl1-1*, which were compared to 40 Col-0 and 40 *tfl1-1* mutant controls. The *tfl1-1* mutant plants showed the canonical phenotype of early flowering, determinate inflorescences, and formation of axillary flowers (Figure 5C,D).

**Figure 7:**
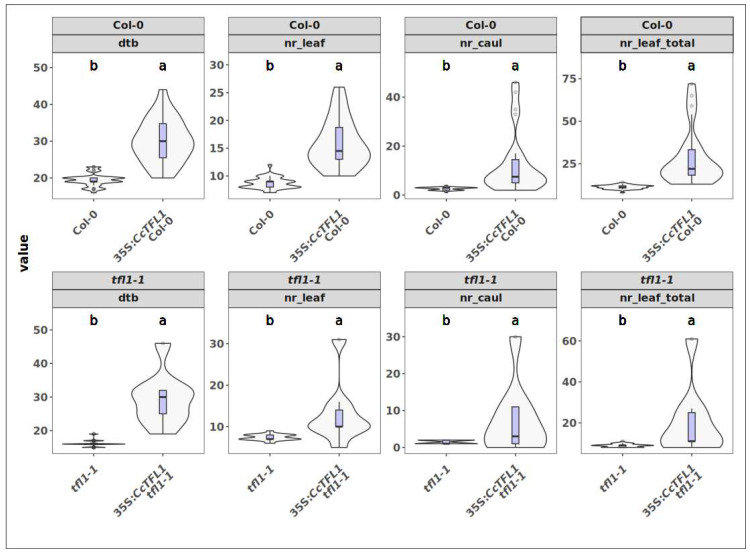
Observations of phenotypic effects of overexpression of *CcTFL1* in Col-0 and *tfl1-1* mutant. Top row. Effects from overexpression of *CcTFL1* in the Col-0 background. Bottom row. Effects from overexpression of *CcTFL1* in the mutant *tlf1-1* background. Phenotypes: “dtb” = days to bolting, “nr_leaf” = maximal number of rosette leaves before bolting, “nr_caul” = number of cauline leaves on the inflorescence, “nr_leaf_total” = total number of leaves. Boxplots inside the violin plots: lines = highest/lowest 1.5× interquartile range, blue boxes = lower/upper quartiles, middle line = median. Compact letter displays summarize results of pairwise comparisons between genotypes.

When *CcTFL1* is overexpressed in Col-0 under the constitutive 35S promoter, 89% of the 35S:*CcTFL1* plants bolted later than the latest Col-0 plant. Bolting time in these plants ranged from 24 days to 44 days, with mode of 30 days (Figure 7, Supplementary Tables S5 and S6). This indicates that *CcTFL1*, when overexpressed, has a strong delaying effect on bolting. Moreover, when compared to Col-0, 35S:*CcTFL1* Col-0 plants had a higher number of rosette leaves as well as cauline leaves.

When *CcTFL1* is overexpressed in *tfl1-1* mutants under the constitutive 35S promoter, 89% of the 35S:*CcTFL1 tfl1-1* plants bolted later than the *tfl1-1* mutant. The bolting time in these plants was in the range of 23-46 days, with a mode of 32 days (Figure 7, Supplementary Tables S5 and S6). Moreover, 88% of the 35S:*CcTFL1 tfl1-1* plants bolted later than the latest-bolting Col-0 individual. This indicates that *CcTFL1* overexpression causes a severe flowering delay. With regard to the number of rosette leaves, leaf numbers in 35S:*CcTFL1 tfl1-1* plants exceeded the number of leaves in the *tfl1-1* mutant. Cauline leaf numbers in 77% of the 35S:*CcTFL1 tfl1-1* plants exceeded those of *tfl1-1*, whereas 56% exceeded the maximum cauline leaf number of Col-0. When considering rosette and cauline leaves together, the numbers remain similar to those for the rosette leaves. Taken together, the results indicate that *CcTFL1* complements the *tfl1-1* mutant and, when overexpressed in wild-type plants, produces the canonical *TFL1* effects such as late bolting and high leaf numbers.

**Figure 8:**
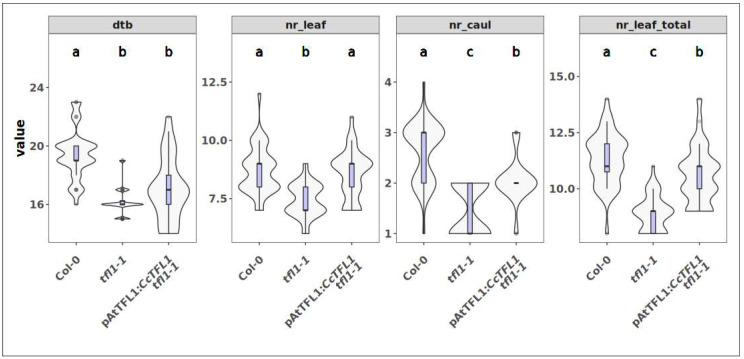
Observations of expression of *CcTFL1* under the Arabidopsis native *AtTFL1* promoter in *tfl1-1* mutant background. Phenotypes: “dtb” = days to bolting, “nr_leaf” = maximal number of rosette leaves before bolting, “nr_caul” = number of cauline leaves on the inflorescence, “nr_leaf_total” = total number of leaves. Compact letter displays summarize results of pairwise comparisons between genotypes.

When *CcTFL1* is expressed under the native Arabidopsis *AtTFL1* promoter, 17% of the pAtTFL1:*CcTFL1 tfl1-1* plants bolted later than the latest-bolting *tfl1-1* mutant and the range of 20–22 das, with a mode of 20 das, overlapping with that of Col-0 (Figure 8). This indicates that *CcTFL1* expression in the T_1_ population complements the early-flowering phenotype of the *tfl1-1* mutant.

The number of rosette leaves in 14% of the pAtTFL1:*CcTFL1 tfl1-1* plants exceeded the number of rosette leaves in the *tfl1-1* mutant whilst within the range of Col-0. Cauline leaf numbers exceeding the maximum number in the *tfl1-1* mutant occurred in 14% of the pAtTFL1:*CcTFL1 tfl1-1* plants, which were within the range of the Col-0 (1-4 leaves). When rosette and cauline leaves are considered together, numbers were similar. Taken together, the results from expressing *CcTFL1* under the native Arabidopsis promoter in the *tfl1-1* mutant background indicate that *CcTFL1* complements the early-bolting, low leaf number, phenotype of this mutant. Despite differences in the magnitude of phenotypic effect when expressed constitutively, or under the native Arabidopsis *TFL1* promoter, our results demonstrate that *CcTFL1* has the canonical flowering-suppressing function reported for *TFL1* in a wide range of plant species.

## 3. Discussion

### 3.1. MFT-Likes from the Campanulid Subclade May Be Subjected to Higher Evolutionary Speed

*MFT*-like genes have been identified in most plant species, suggesting that they have a critical function. The presence of a distinct subclade encompassing members from the Campanulids (Asteraceae and Apiaceae families) within the *MFT1* clade has, to our knowledge, not been reported before. This can most likely be attributed to earlier phylogenetic studies on the evolution of the PEBP family not including taxa from Campanulids, such as in Hedman et al., 2009 [30] and Bennet and Dixon, 2021 [38]. We have not found a clear explanation for this apparent increased rate of evolution, but we speculate that it is linked to a potential loss of function in this clade, which removes the need for sequence conservation. This is supported by the fact that the Campanulids under study have accumulated significant numbers of mutations in regions that are otherwise highly conserved in other taxa (Supplementary Figure S4).

### 3.2. Functional Conservation of CcFT Is Not Fully Certain

*FT* homologs have been identified in multiple plant species, including in Asteraceae. In lettuce, mRNA from *LsFT* was found to be abundant in the oldest leaves under flowering-inductive controlled high-temperature conditions, and was also detected during the vegetative-to-reproductive phase transition [63,72]. Overexpression caused early flowering in wild-type Arabidopsis, although not to the extent of *AtFT* [63]. It could, however, fully complement the late-flowering phenotype of the *ft-2* mutant [73]. Moreover, knockdown of *LsFT* by RNA interference-delayed bolting [73]. These results indicate that *LsFT* is a functional homolog of *AtFT* and a regulator of bolting in lettuce. In other Asteraceae, multiple *FT* homologs have been reported. Sunflower (*Helianthus annuus* L.) contains four FT paralogs, numbered *HaFT1* through to *HaFT4*, with *HaFT1* being a repressor and *HaFT4* a promoter of flowering [68]. In *Chrysanthemum* sp., three *FT* homologs exist, *CsFTL1*, *CsFTL2*, and *CsFTL3*, although under flowering inductive SD conditions, only the latter is upregulated. Under non-inductive conditions such as heat, *CsFTL3* is downregulated, whereas overexpression of the gene in Chrysanthemum is capable of inducing flowering under non-inductive conditions, suggesting it to be a floral activator [69,74]. Globe artichoke, like lettuce, possesses one *FT* homolog, although *CcFT* is apparently not expressed significantly before the vegetative-to-reproductive phase transition, as is the case for *LsFT* [63]. *CcFT* expression instead starts to increase once inflorescences start developing. This difference in timing, in addition to *CcFT* not being capable of rescuing the *ft-10* phenotype, may suggest a difference in function of *CcFT* in globe artichoke. The latter may be linked to the mutation we identified in the LYN triad on exon 4, which is critical to *FT* function. An example of neofunctionalization of *FT* was given by Pin et al. [60] for beet (*Beta vulgaris*). In this species, a duplication of the *FT* gene, followed by mutations in a critical domain, resulted in flowering being under the control of an antagonistic pair of *FT* genes, one being a flowering promoter, and one being a flowering repressor. However, an alternative explanation for our finding may be that multiple copies of *CcFT* occur on the genome that have hitherto not been resolved. This can, for example, be the case if a gene has multiple copies with high sequence similarity that cannot be separated during genome assembly. No genomic sequence variation in *CcFT* was, however, found in resequencing data during this study. Such variation would have been more likely if multiple copies of the same genes have been collapsed on the reference genome. Another possibility is that one or more homologs of *FT* exist in globe artichoke for which genomic sequences have become too dissimilar from *FT* query sequences for BLAST analysis. This may be the case if, for example, large introns exist in one or more of the homologs. The problem of separating multicopy genes may be addressed by performing BLAST analyses using smaller query sequences, for example, one exon in size. Alternatively, FISH may be used to detect multiple copies if their physical separation is sufficient.

Together with the B segment, the LYN triad on exon 4 is essential for the *FT* protein to function, although reports on the effects of mutations in this triad have been contradictory. Examples of *FT* genes to which mutations in the B segment and LYN triad of exon 4 do not confer flowering repressive effects are *PsFTc* (*Pisum sativum* L.) [75], *ZCN8* in corn (*Zea mays* L.) [76], and *HvFT3* (*Hordeum vulgare* L.) [77]. On the contrary, examples of *FT* genes that acquired flowering repressing functions from mutations in said regions are *BvFT1* (*Beta vulgaris* L.) [27], *SP5G* in tomato (*Solanum Lycopersicon* L. [78], and *CsatFT4* in crocus (*Crocus sativus* L.) [79]. These species harbor multiple paralogs of the *FT* gene, with at least one always retaining its flowering-promoting effect. It is, therefore, possible that the single homolog of *FT* in globe artichoke, *CcFT*, is a functional flowering promoter despite harboring an Ala149 mutation in the LYN triad of the protein. On one hand, the mutation did not appear to significantly change the structure of the *CcFT* protein when folding was simulated in silico. On the other hand, *CcFT*, when overexpressed in the late-bolting, high-leaf number *ft-10* mutant, neither resulted in early-bolting plants, nor complemented the mutant phenotype. Moreover, the effect of *CcFT* was particularly limited when compared to that of overexpressing *AtFT* from Arabidopsis. Although more research would be needed to definitely determine the functionality of *CcFT* in globe artichoke, our findings leave open the interesting possibility that the flowering-promoting effect of *FT* may not be a major floral integrator and promoter of the floral transition in this species.

Expression of *CcFT* reaches high levels in the leaves after the vegetative-to-reproductive phase change. This contrasts to Chrysanthemums, wherein *FT*-like genes are predominantly expressed in the leaves, with a peak around the moment of the vegetative-to-reproductive phase and high levels thereafter [69,80]. Moreover, the results are also not fully in line with reports from lettuce, where expression in the largest leaf increases during the vegetative-to-reproductive phase transition and continues to increase during subsequent inflorescence development [72]. Nevertheless, in the same study, *LsFT* levels varied between the two bolting cultivars tested. In the early bolting variety ’Leaf Lettuce Green’, expression remained close to zero, even as doming already commenced (which is the earliest sign of bolting in lettuce), and was followed by a large increase during the development of the inflorescence. In later bolting cultivar, ’TG’ expression followed a similar pattern, although expression before doming increased slightly with time. Given that *CcFT* in globe artichoke is only significantly expressed in the leaves at pre-bolting stage 3, when the primary inflorescence is already developing, this might suggest that *CcFT* has acquired a function in inflorescence and/or flower development, being less relevant in the control of the vegetative-to-reproductive phase transition.

Both in the *ft-10* and in the Col-0 background, overexpression of *CcFT* had a weaker effect on bolting time and the number of rosette leaves than *AtFT*. Col-0 flowered about two days earlier than 35S:*CcFT* Col-0 and five days later than 35S:*AtFT* Col-0. 35S:*CcFT ft-10*, and was not able to fully complement *ft-10*. A somewhat similar observation has been described for *LsFT* from lettuce by [63], where Col-0 (30 days) bolted 2–4 days later than 35S:*LsFT* Col-0 (26–28 days), whereas 35S:*AtFT* Col-0 (20–23 days), likewise, bolted even earlier. This was interpreted as *LsFT* being capable of partially complementing the phenotype of the *ft-10* mutant. At the protein sequence level, LsFT and AtFT have a similarity of 74.3%, whilst LsFT and CcFT are 94.3% similar. Possibly, the observations of partial complementation of *CcFT* and *LsFT* could be associated with this high level of genetic similarity, although *HsFT1* from the related sunflower is able to fully restore the phenotypic defect in *ft* [62].

### 3.3. Homologs from the TFL1-Like Clade Are Conserved Between Asteraceae

*TFL1/CEN/BFT*-like genes have been identified in Asteraceae and the number of homologs varies between species. In this study, we identified three *TFL1/CEN* and two *BFT* homologs in globe artichoke. The three *TFL1/CEN* homologs are closely related to *CsTFL1* and *CsCEN*-like from *Chrysanthemum seticuspe*, for which reason we decided to maintain the naming from that species. It must be remarked however that the distinction between *TFL1* and *CEN* is difficult to make, and this leaves open the possibility that the distinction in globe artichoke turns out to be arbitrary after more data from Asteraceae become available. *CsCEN*-like was found to be highly expressed in vegetative apices in plants under non-flowering-inductive conditions, and it was proposed that it might be involved in regulating flowering competence in relation to seasonal changes [67]. The expression in vegetative shoot apices is similar to what we have observed for *CcCENa*, which, in conjunction with high sequence similarity, raises the possibility of *CcCENa* having a similar function in globe artichoke. *CsTFL1* is expressed mainly in the root and shoot tips [47], whereas *CcTFL1* levels are high in the former but not in the latter. Taken together, the results from this study indicate that *CcTFL1* is a homolog of *AtTFL1*. Concerning the identity of *CcCENa* and *CcCENb*, more data would be needed to determine whether these genes have evolved into proper *CEN/ATC* orthologs, or whether they are just paralogs of *CcTFL1* resulting from a relatively recent genome duplication event.

Two globe artichoke PEBPs were found to be more related to the BFT protein. In Arabidopsis, *BFT* functions as a repressor of flowering and is mainly expressed in the SAM, young leaf, and axillary inflorescence meristem [50]. This expression pattern is mostly in line with that of *CcBFTb*. The other homolog, *CcBFTa*, is missing several motifs, in a way suggestive of a deletion, and is only expressed in the hypocotyl, imbibed seed, and cotyledon. This suggests that *BFT* is conserved in globe artichoke, although functional characterization would be required to confirm this.

*CcCENa* is expressed before the vegetative-to-reproductive phase transition. This is opposed to the expression of *CcTFL1*, which is highest predominantly after the vegetative-to-reproductive phase transition. These abrupt changes in expression would qualify *CcCENa* and *CcTFL1* for the development of molecular markers to determine the moment of this transition in subsequent studies. Changes in expression of *CcBFTa* and *CcBFTb* are less pronounced, rendering them less suitable candidates for this purpose. The development of molecular markers would, however, require more detailed study on the time and magnitude of expression changes, the detailed dynamics of this expression in the vegetative and reproductive phases, and the cosegregation of those markers with bolting in known early- and late-bolting genotypes.

In this study, we characterized genes belonging to the PEBP family in globe artichoke with the objective of gaining more insight into the genetic architecture of the vegetative-to-reproductive phase change in this species. We found that the seven PEBP family members were mostly conserved between this species and related Asteraceae such as lettuce, chrysanthemum, and sunflower. There are indications that the *MFT*-likes family in Asteraceae, and possibly the Campanulids clade, underwent a change in the speed of evolution. Moreover, we showed that *CcTFL1* is expressed in the shoot apex and developing inflorescences and is able to complement the *tfl1* mutant, whilst *CcFT* expression is upregulated in the shoot/inflorescence apex after the floral transition and only partially complements the *ft-10* mutant. This suggests that the function of *CcTFL1* is conserved whereas the function of *CcFT* is not fully conserved.

## 4. Materials and Methods

### 4.1. Phylogenetic Study and Gene Characterization

Sequences from Arabidopsis PEBP family member proteins FT (AT1G65480.1), TSF (AT4G20370.1), TFL1 (AT5G03840.1), BFT (AT5G62040.1), ATC (AT2G27550.1), and MFT (AT1G18100.1) were downloaded from the TAIR website [81]. BLASTp was performed in CLC Genomics Workbench v21.0.3 (QIAGEN, Aarhus, Denmark) withBLAST+ version 2.6.0 with an E-value cutoff of 10^−5^ and no masking of low-complexity regions. The database that was BLASTed against was constructed from the protein sequences of 28,632 gene models that were downloaded from the Hi-C v2 genome dataset on the Globe Artichoke Genome Database website [71,82]. Protein domains were predicted by HMMER 3.1b.

For the gene phylogeny study, homologous genes from different species were acquired from the phytozome [83] and NCBI websites (https://www.ncbi.nlm.nih.gov, accessed on 30 September 2022). Additional *MFT1*-like and *MFT2*-like sequences were acquired from Bennett and Dixon, 2021 [38]. The alignment of protein sequences, and calculation of phylogenetic trees, were performed with MUSCLE [84,85] with default settings. Genetic distances were calculated with the dist.ml function implemented in Phangorn [86] using a WAG amino acid replacement matrix [87]. The initial NJ tree was inferred though the BIONJ algorithm implemented in the ape package [88,89]. For the ML tree, MODELTEST [90] was used to find the optimal substitution model, which was subsequently used to calculate the ML tree and bootstrap values with Phangorn (1000 bootstraps, optNni = T). Maximum parsimony was inferred with Phangorn using the pratchet function (minit = 1000, edge lengths according to accelerated transformation (ACCTRAN) algorithm). The resulting tree was drawn with ggtree [91]. Amino acid sequence similarities were calculated by their pairwise alignment as 100 × (identical positions)/(aligned positions + internal gap positions). Multiple sequence alignments were performed in CLC Genomics Workbench.

Conserved motifs were identified by analyzing the full length PEBP sequences using the Multiple Expression motifs for Motif Elicitation algorithm [92] as available in the MEME suite 5.5.0 [93] with the following parameters: maximum nr of motifs = 11, minimum motif width = 4, max motif width = 50, and minimum nr of sites = 4. Motifs were visualized with the R package ggmotif [94].

### 4.2. Expression of PEBP Orthologues

Primers were designed with CLC Genomics Workbench (Supplementary Table S2). Reference genes were *CcACT7* and *CcETIF1a*, the latter adapted from a study in sunflower by [95]. Amplicons were tested by high-resolution melting curve (HRMC) analysis on magnetic induction cycler (mic) qPCR system (Bio Molecular Systems, Upper Coomera, QLD, Australia). For *CcCENb* and *CcMFT,* no pure amplicons could be produced after testing three different primer designs and these genes were thus excluded from further analysis.

Individuals from two genotypes, one early and one late bolting, were sown in a nursery in late July 2020 and transplanted to a net house in the vicinity of Águilas, Spain, in September. The globe artichoke genotypes used in this study were provided by BASF‘s vegetable seeds business (Valencia, Spain). Developmental stages of the plant and shoot apices were scored according to the scale described in Berentsen et al., 2024 [13]. The “Imbibed_seeds” sample comprised a pool of three grinded seeds from the late bolting genotype that had been imbibed in the dark at ambient temperature on wetted tissue for 72 h. The sample “Cotyledon” was a pool of two ±0.5 cm long cotyledons sampled 18 das from plants in an 8 h light/16 h dark photoperiod. Tissues sampled at the net house, comprising three technical replicates for each genotype, were stored in μL RNAlater™ solution (Thermo Fisher Scientific, Waltham, MA, USA) and stored at −18 °C. Tissues sampled from seeds, hypocotyls, and cotyledons were stored at −80 °C. RNA was extracted with the EZNA^®^ Plant RNA kit (Omega Bio-tek, Norcross, GA, USA) with on-column DNAse treatment with an RNase-free DNase I Set (Omega BioTek, USA). RNA concentrations were measured on a NanoDrop 1000 spectrophotometer (Thermo Fisher, USA) and normalized prior to cDNA synthesis with a SuperScript IV First-Strand Synthesis System (Thermo Fisher, Waltham, MA, USA). qPCR reactions were performed on a magnetic induction cycler (mic) qPCR system (Bio Molecular Systems, Australia) in a 10 μL reaction volume with the following composition: 1× PyroTaq EvaGreen reaction mix with ROX (Cultek Molecular Bioline, Madrid, Spain), 0.125 mM of each primer, and 20 ng cDNA. Melting curve analyses were performed to ensure the specificity of the amplicon.

### 4.3. Production of Entry Clones

Primers (Supplementary Table S2) were designed for coding sequences of *CcFT* and *CcTFL1*, which were subsequently amplified from an inflorescence primordium sample taken from a proprietary commercial hybrid parent clone provided by BASF’s vegetable seeds business, in an unheated nethouse 150 days post transplanting. The sample was stored at −18 °C in 300 μL RNAlater™ solution (Thermo Fisher Scientific, USA) until RNA extraction with the EZNA^®^ Plant RNA kit (Omega Bio-tek, USA) with on-column DNAse treatment with the RNase-free DNase I Set (Omega BioTek, USA). cDNA was synthesized with a SuperScript IV First-Strand Synthesis System (Thermo Fisher, USA) according to the manufacturer’s instructions. *CcFT* and *CcTFL1* target CDSes were amplified with Phusion HF polymerase (NEB, Rowley, MA, USA) and amplicons resolved by electrophoresis on a 1.5% agarose gel for by band-cut-out (BCO) before being purified with the Zymoclean Gel DNA Recovery Kit (Zymo Research, Irvine, CA, USA). Then, 3′ overhangs were added, followed by purification of the product with the Metabion miPCR purification kit (Metabion International, Planegg, Germany). Amplicons were cloned into the pCR™8/GW/TOPO^®^ destination vector (Invitrogen, Carlsbad, CA, USA) and the cloning reaction was dialyzed prior to transforming One Shot^®^ TOP10 Chemically Competent Escherichia coli (Invitrogen, USA). The CDS of *AtFT* was amplified from a pDONR201:*AtFT* entry vector in the same fashion as described for *CcFT* and *CcTFL1* and cloned into a pDONR207 entry vector (Thermo Fisher, USA).

### 4.4. Production of Expression Vectors

Expression vectors were produced by Gateway recombinatorial cloning (Invitrogen, USA) of the three aforementioned entry clones and pEARLEYGATE100 donor vectors (Thermo Fisher Scientific/Invitrogen, USA). In addition, *CcTFL1* was cloned into the pTFL1 expression vector, which combines a native *AtTFL1* promoter with selective markers DsRed [96] and phosphinothricin ammonium (commercial name “BASTA”) resistance [97]. This vector contains all the regulatory sequences previously reported by Serrano-Mislata et al., 2016 [98]. Since entry vector pCR8/GW/TOPO:*CcTFL1* and pTFL1 shared a common selective antibiotic resistance, the former was linearized with XhoI and dephosphorylated with rapid alkaline phosphatase (Sigma-Aldrich, Saint Louis, MO, USA) in 1 x rapid alkaline phosphatase buffer (Sigma-Aldrich, USA) prior to the LR cloning reaction. To overcome low LR reaction efficiency between pCR8/GW/TOPO:*CcFT* and pEARLEYGATE100, the former was linearized with XbaI and dephosphorylated. LR Gateway recombinatorial cloning reactions were performed with ~150 ng entry vector and ~150 ng expression vector, with 2 μL LR clonase II enzyme mix (Invitrogen, USA), filled up to a reaction volume of 10 μL, dialyzed, and used to transform OneShot TOP10 chemically competent E. coli cells (Invitrogen, USA), or electrocompetent EZ10B E. coli in the case of pEARLEYGATE100:*AtFT*. Expression clones were confirmed by restriction analysis with PstI/EcoRI for pEARLEYGATE100:*CcFT* and pEARLEYGATE100:*AtFT*, SalI for pEARLEYGATE100:*CcTFL1* and pTFL1:*CcTFL1*.

### 4.5. Transformation, Growing, and Observation of Transgenics

Binary expression vectors were transferred to Agrobacterium tumefaciens C58C1 RIFT (pMP90) competent cells. Col-0 and *ft-10* [56] were grown under LD and tfl1-1 [45] under SD conditions prior and after transformation according to the floral dip method by [99]. T_1_ transgenic seeds of pTFL1:*CcTFL1 tfl1-1* were sown individually in 5 cm square pots, while seeds from the remainder genotypes were treated with a selective medium consisting of 50 μg/mL phosphinothricin ammonium (Duchefa Biochemie B.V., Haarlem, The Netherlands) and 0.05% Silwet-77 (Plant Media, Dublin, OH, USA) prior to being moved to the LD greenhouse. The plants were observed in 4–7-day intervals. Statistical analyses on the phenotypic observations from T_1_ plans were performed by ANOVA and the R-package “emmeans” [100].

## Data Availability

The raw data supporting the conclusions of this article will be made available by the authors on request.

## Supplementary Materials

The following supporting information can be downloaded at: https://www.mdpi.com/article/10.3390/plants14091364/s1, Supplementary Figure S1. Additional dendrograms from MFT protein sequences. Supplementary Figure S2. Gene structure of PEBP members in globe artichoke. Supplementary Figure S3. Seqlogos from MeMe motif search in PEBP proteins from different plant species. Supplementary Figure S4: Protein alignments of selected *MFT*-likes and Alphafold 3D protein models of *CcFT* and *AtFT.* Supplementary Table S1. PEBP gene homologues in Cynara cardunculus. Supplementary Table S2. qPCR primers. Supplementary Table S3. Cloning primers.Supplementary Table S4. Tissues sampled for the study of gene expression. Supplementary Table S5. Overexpression of CcFT and CcTFL1 and complementation of ft-10 and tfl1-1 mutants. Supplementary Table S6. Phenotypic distributions in T1 lines.

## Abbreviations

The following abbreviations are used in this manuscript:

FT	FLOWERING LOCUS T
TFL1	TERMINAL FLOWER 1
dtb	Days to bolting
das	Days after sowing
