# Supplementary material for: Assessing Functional Conservation Amongst FT- and TFL1-like Genes in Globe Artichoke
Source: Plants (Basel). 2025 Apr 30;14(9):1364. doi: 10.3390/plants14091364 (PMC12073138; doi:10.3390/plants14091364)
Supplement: Supplementary file 1 [file plants-14-01364-s001.zip › plants-3539814-supplementary.pdf]

Supplementary Figures

A

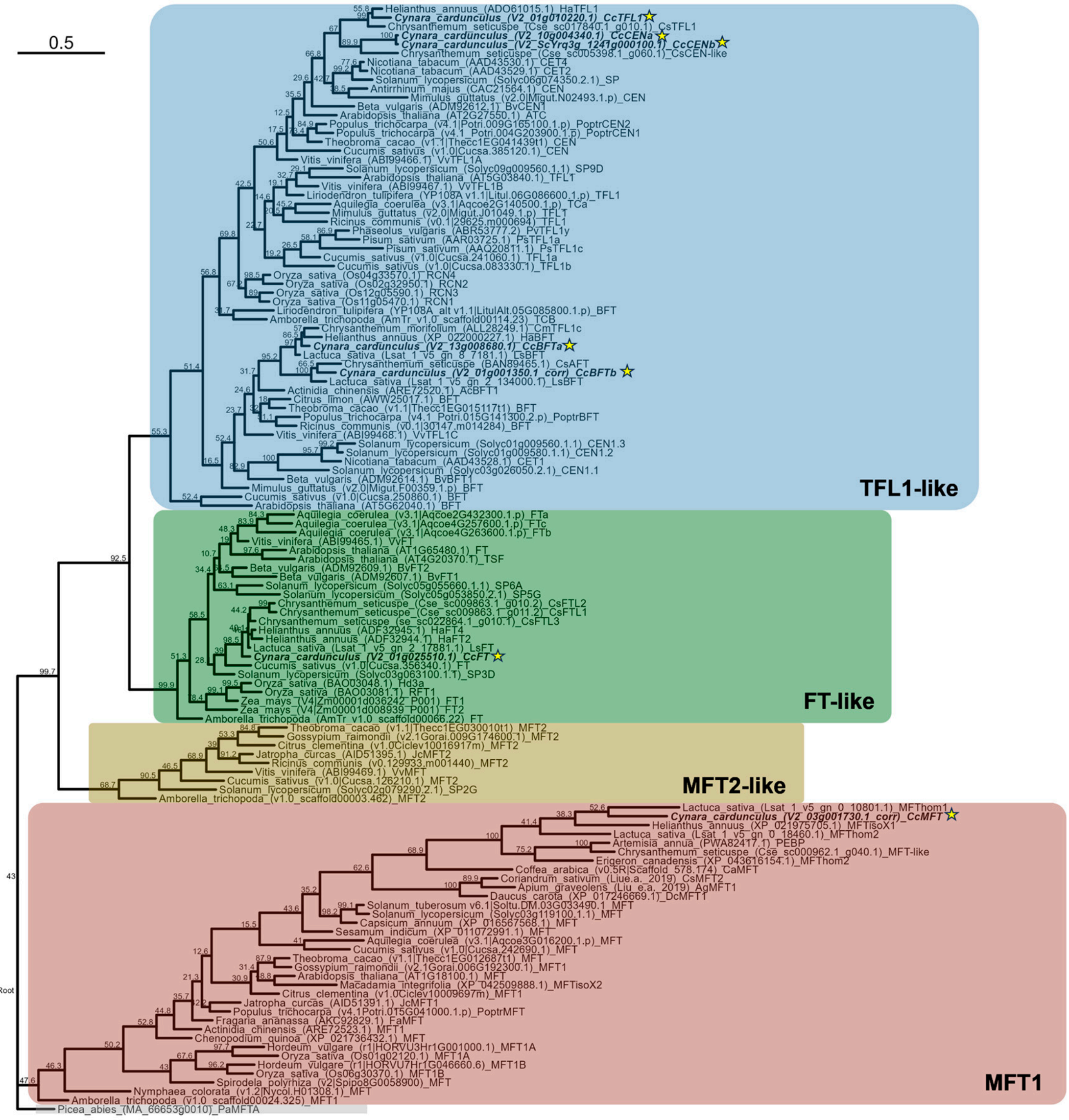

B

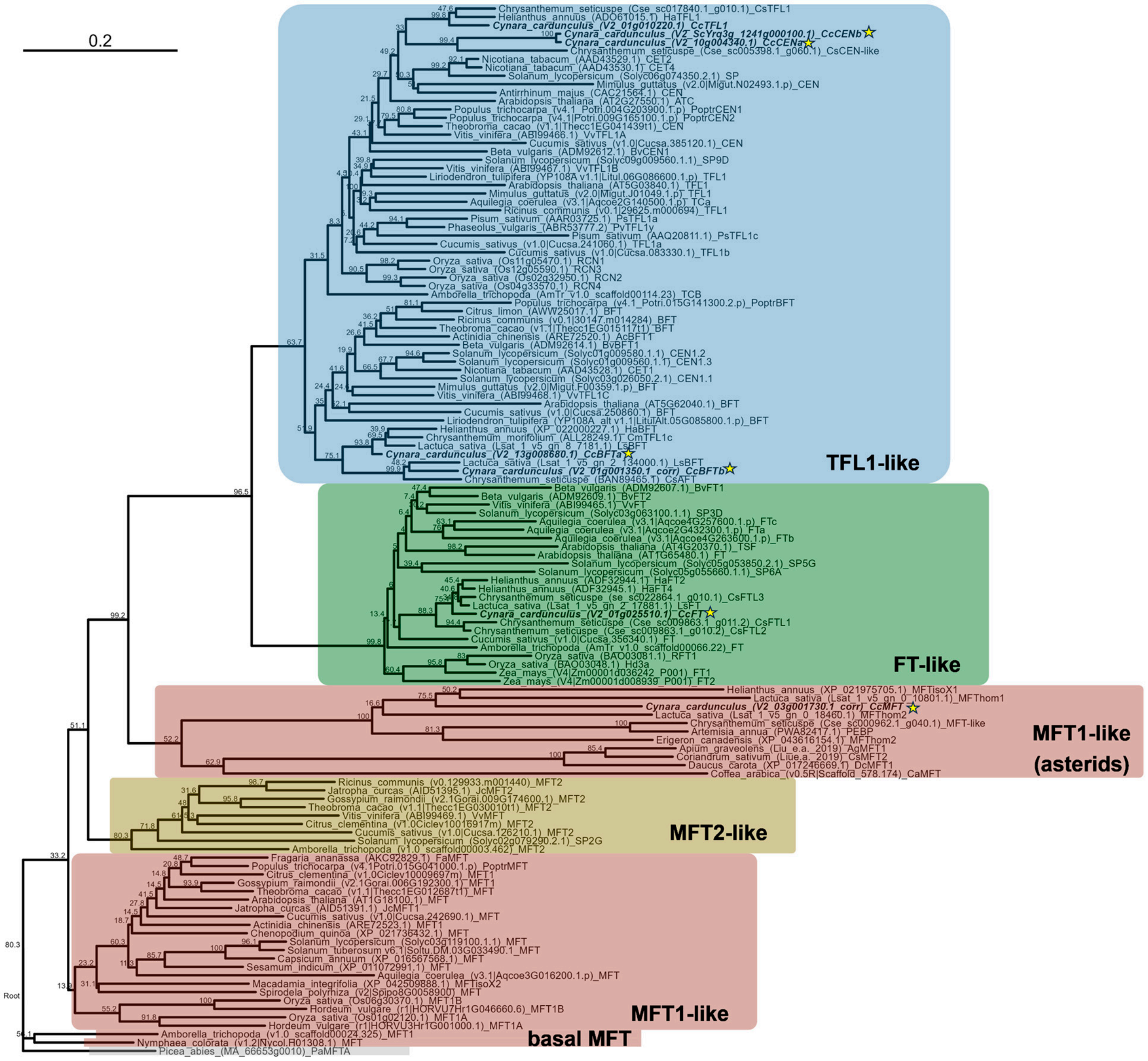

Supplementary Figure S1. Additional dendrograms from MFT protein sequences. A. Maximum Parsimony (MP), B**.** Neighbor Joining (NJ). PEBP members from globe artichoke in bold and marked with a star. Node text represents 1000-bootstrap values.

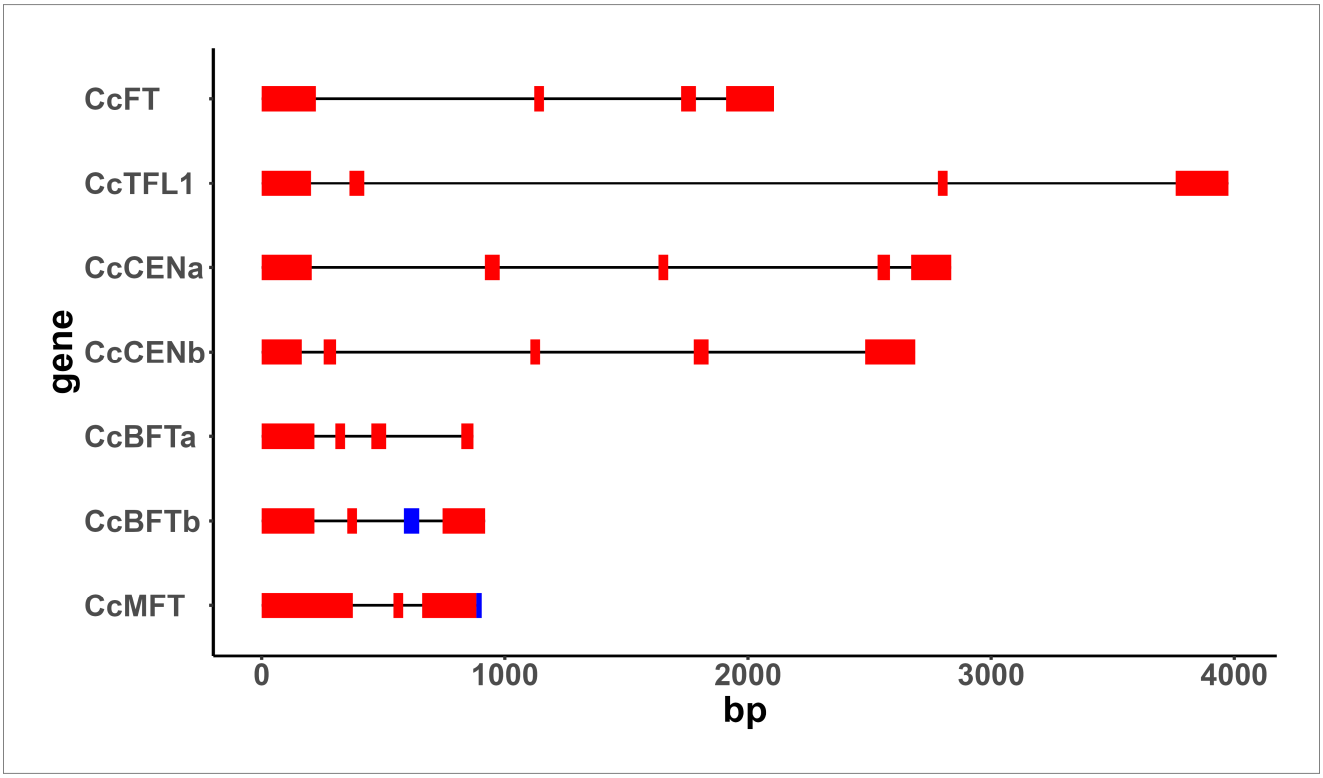

Supplementary Figure S2. Gene structure of PEBP members in globe artichoke. Black lines = genomic sequence of gene model, red boxes = exons, blue boxes = exons after correction.

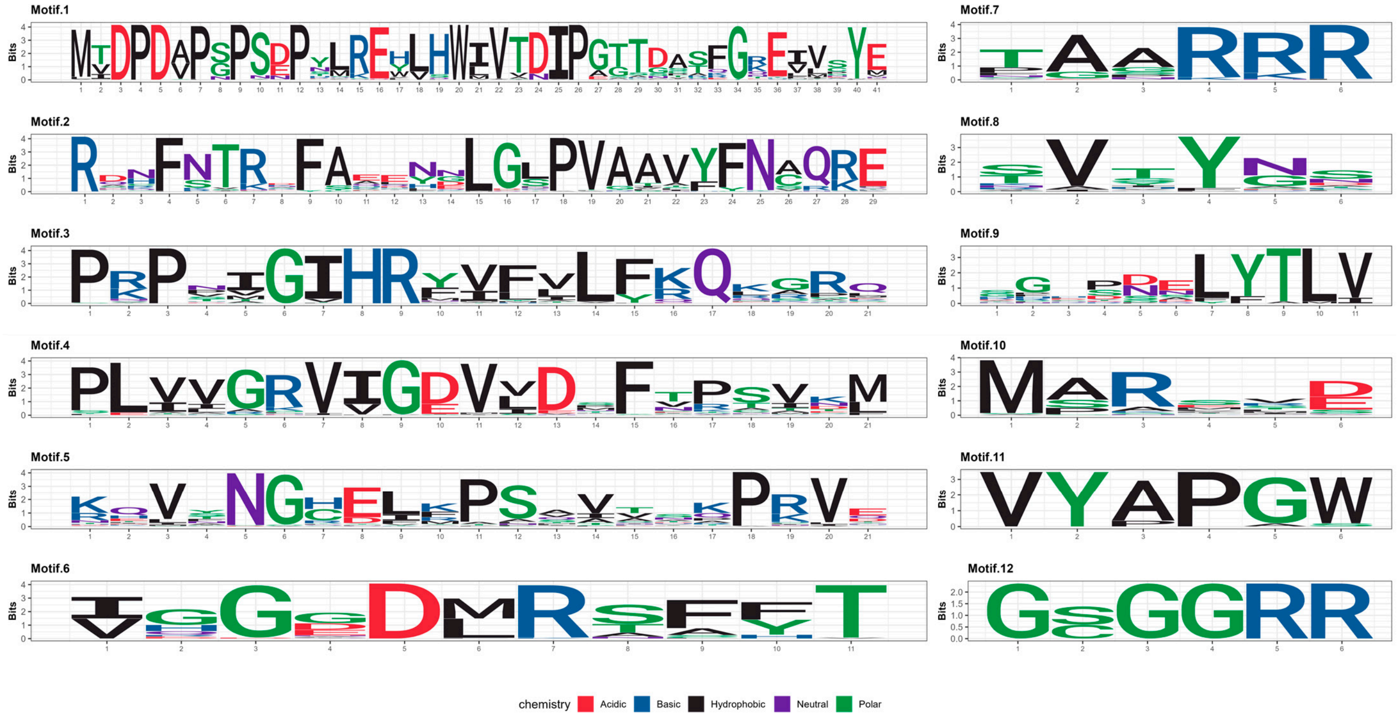

Supplementary Figure S3. Seqlogos from MeMe motif search in PEBP proteins from different plant species.

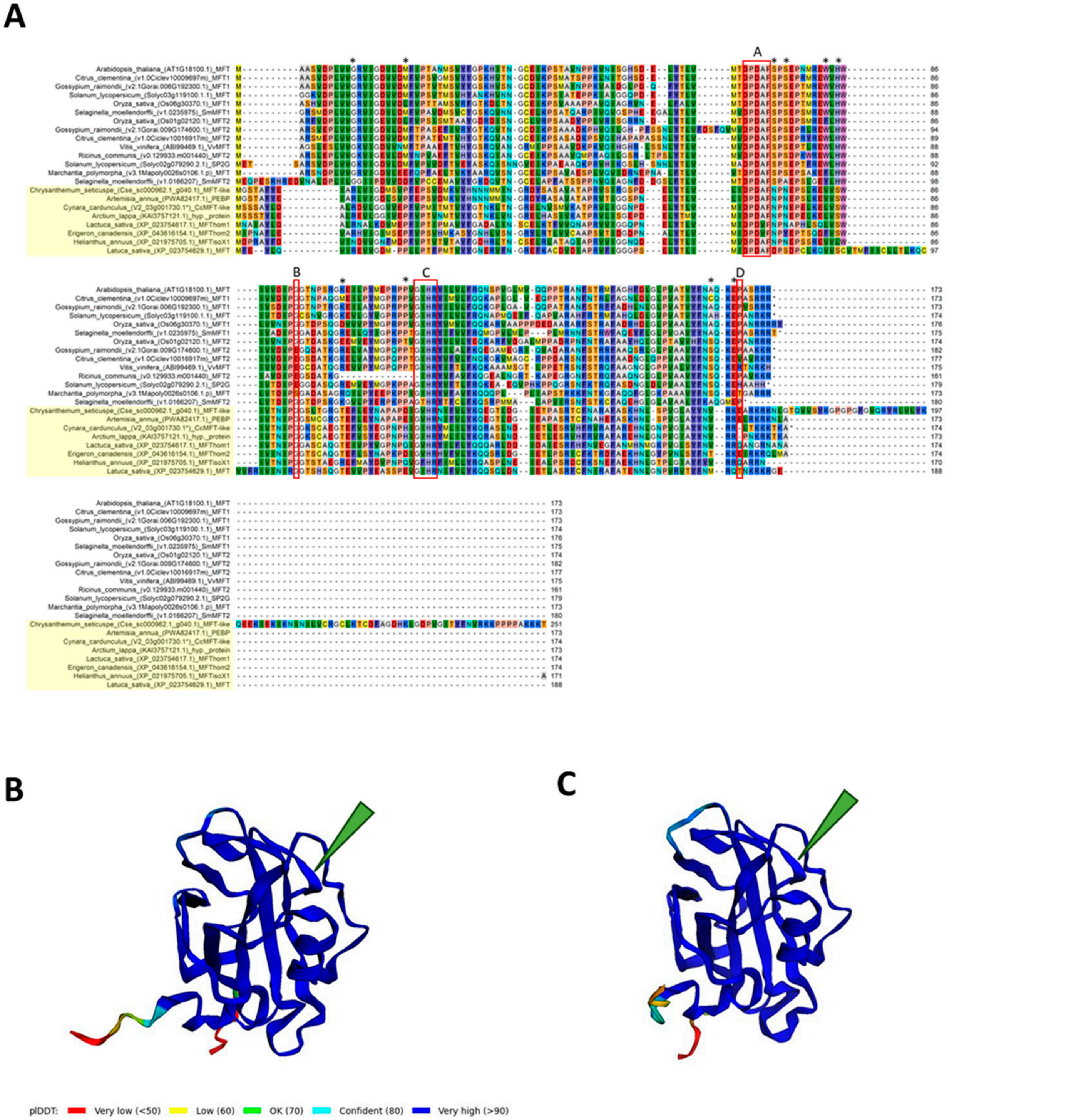

Supplementary Figure S4: Protein alignments of selected *MFT*-likes and Alphafold 3D protein models of *CcFT* and *AtFT*. **A.** *MFT*-likes. Red boxes denote motifs: A = D-P-D-x-P motif, B = Gly/Glu residue linked to *MFT1*/*MFT2* clade respectively, C = G-x-H-R motif, D = Proline residue unique to *MFT*-likes, * = Residues being substituted in at least 6/7 Asteraceae *MFT*-likes. Asteraceae emphasized by yellow squares. **B.** Alphafold 3D model of *CcFT*. **C)** Alphafold 3D model of *AtFT*. Green arrows in figures 2B and 2C point to the first amino acid of the LYN triad.

Supplementary Tables

Supplementary Table S1. PEBP gene homologues in Cynara cardunculus

Name	GeneID	Length (a.a.)	domains[a]	Genomic position	CDS length	number of exons predicted[b]	number of exons corrected[c]	Remarks
CcFT	V2_01g025510.1	174	PBP	Chr_01:48409302-48411409	525	4		
CcTFL1	V2_01g010220.1	174	PBP	Chr_01:11496205-11500181	525	4		
CcCENa	V2_10g004340.1	175	PBP	Chr_10:4603742-4606578	528	5		Exon 4 from AtBFT split into two exons
CcCENb	V2_ScYrq3g_1241g000100.1	175	PBP	ScYrq3g_1241:3332-6020	528	5		Exon 4 from AtBFT split into two exons
CcBFTa	V2_13g008680.1	123	PBP	Chr_13:27752375-27753246	372	4		
CcBFTb	V2_01g001350.1	144	PBP	Chr_01:1105026-1105945	435	3		Orginal gene model
	V2_01g001350.1_corr	168	PBP	Chr_01:1105026-1105945	507		4	Added exon 2
CcMFT	V2_03g001730.1	204	PBP	Chr_03:1528750-1529633	641	3		Orginal gene model
	V2_03g001730.1_corr	211	PBP	Chr_03:1528750-1529633	636		3	22 a.a. added to end of exon 3

[a] Significant Pfam domains
[b] Exons predicted, the nr of exons according to the gene model by (Acquadro, Portis, Valentino, Barchi, & Lanteri, 2020) [64]
[c] Exons corrected, number of exons after manual curation of RNA sequences

Supplementary Table S2. qPCR primers

target	primer name	sequence	F/R[a]	Tm (°C)
CcFT	ORB_15	AGCTACGACCCTCTCAGGTT	F	57.5
	ORB_16	TCGGGATCCACCATGACTAAAG	R	56.3
CcTFL1	ORB_17	TGGCATCCACAGGTTTGTGT	F	57.3
	ORB_18	GGGCAAGTAACAGTCTGCCT	R	57.4
CcCENa	ORB_23	CATACCAGGCACCACTGACA	F	57.1
	ORB_24	CCTGGCCTTGGCATCTCATA	R	57.1
CcBFTa	ORB_31	CTCCACCAGCTTCCAGAGAC	F	57.3
	ORB_32	ACAGCAGCAACTGGTAACCC	R	57.9
CcBFTb	ORB_95	CAAAGAGCTAGGCAATCA	F	49.5
	ORB_96	AGCAACTGGTAACCCTAA	R	50.4
CcMFT-like	ORB_93	TCCGAACCCAAATGAGCC	F	55.3
	ORB_94	TTCAGCACACGACTTCCC	R	55.1

[a] Forward (F) or reverse (R) primer

Supplementary Table S3. Cloning primers

target	accession (CDS)[a]	sequence[b]	F/R[a]	Tm (°C)	amplicon length (bp)
CcFT	XM_025120041	ATGATGCCTAGGGAGAGGGA	F	57.1	528
		TTATCTCCGTCGTCCACCGA	R	57.6	528
CcTFL1-A	XM_025121321	ATGGCAAGAATCACGTCAGATCCTCTT	F	59.8	587
		TTAACGTCTTCTGGCTGCAGTTTCCC	R	61.1	587
AtFT	AT1G65480.2	**GGGGACAAGTTTGTACAAAAAAGCAGGCT**ATGTCTATAAATATAAGAGACC	F	63.7	586
		**GGGGACCACTTTGTACAAGAAAGCTGGGT**CTAAAGTCTTCTTCCTC	R	66.7	586

[a] accession used for primer design
[b] bold nucleotides in sequence denote attB overhangs

Supplementary Table S4. Tissues sampled for the study of gene expression.

sample name short[a]	description	genotype	developmental stage	d.p.t.[b]
SAM_stage_0	Dissected SAM from individual in stage 0	late	pre-bolting stage 0	63
SAM_stage_1	Dissected SAM from individual in stage 1	late	pre-bolting stage 1	147
SAM_stage_3	Dissected SAM from individual in stage 3	late	pre-bolting stage 3	147
Leaf_stage_0	Punch from yongest mature leaf from individual in stage 0	late	pre-bolting stage 0	63
Leaf_stage_1	Punch from yongest mature leaf from individual in stage 1	late	pre-bolting stage 1	147
Leaf_stage_3	Punch from yongest mature leaf from individual in stage 3	late	pre-bolting stage 3	147
Petiole	Transverse section of youngest mature leaf petiole	early	pre-bolting stage 0	56
Center_infl_stem	Pith of primary inflorescence stem	early	bolting_stage_2	147
Cortex_infl_stem	Cortex of primary inflorescence stem	early	bolting_stage_2	147
Cauline_leaf	Cauline leaf (±20 cm, mature)	early	bolting_stage_2	147
Stem base	Radial longitudinal section of stem at base	early	pre-bolting stage 0	56
Stem_below_SAM	Radial longitudinal section of stem below SAM	early	pre-bolting stage 0	56
Immature_head	Radial longitudinal of secondary head /±2 cm in length)	early	harvest stage 1	147
Young_bract	Outer bract from developing primary inflorescence (Ø +/- 5 cm)	late	bolting stage C	167
Mature_bract	Outer bract from primary head at harvest stage (Ø +/- 15 cm)	early	harvest stage 1	147
Receptacle	Receptable of primary inflorescence shortly after anthesis	early	anthesis stage 1	167
Ovary	Ovary of floret	early	anthesis stage 2+	167
Stigma	Purple stigmas (±1 cm in length), close to being receptive	early	anthesis stage 2+	167
Root_tip	Tip of root	early	pre-bolting stage 0	56
Imbibed seeds	Pool of three 72h imbibed seeds	late (seed)		3
Cotyledon	Cotyl (pool of cotyledons ±0.5 cm in length)	late (seed)		18

[a] sample name as used in this paper

[b] "D.p.t." = Days post transplanting, days post start of imbibement for "Imbibed_seeds" and "Cotyledon"

Supplementary Table S5. Overexpression of *CcFT* and *CcTFL1* and complementation of *ft-10* and *tfl1-1* mutants.

				dtb		nr rosette leaves		nr cauline leaves		total nr leaves	
exp.[a]	genotype	construct	n[b]	av	sd	av	sd	av	sd	av	sd
ft	Col-0	(wt)	20	18.0	1.0	9.8	1.1	2.7	0.7	12.5	1.6
ft	Col-0	35S:CcFT	20	19.5	2.2	11.3	2.2	2.9	0.8	14.2	2.8
ft	Col-0	35S:AtFT	20	13.5	2.4	4.3	1.7	2.3	1.0	6.6	2.2
ft	ft-010	(wt)	19	29.8	1.8	28.4	2.4	7.9	0.7	36.3	2.9
ft	ft-010	35S:CcFT	18	29.6	2.5	24.7	3.3	6.4	1.5	30.9	4.4
ft	ft-010	35S:AtFT	19	12.4	2.7	4.1	1.1	1.9	1.1	6.1	1.9
tfl1	Col-0	(wt)	40	19.5	1.7	8.7	1.0	2.6	0.6	11.3	1.2
tfl1	Col-0	35S:CcTFL1A	26	30.0	5.9	15.8	4.3	12.4	12.4	28.2	16.5
tfl1	tfl1-1	(wt)	40	16.2	1.0	7.4	0.7	1.5	0.5	8.9	0.8
tfl1	tfl1-1	pAtTFL1:CcTFL1A	29	17.1	2.2	8.6	1.1	2.1	0.5	10.6	1.3
tfl1	tfl1-1	35S:CcTFL1A	9	29.3	7.8	13.0	7.4	7.2	9.6	20.2	16.8

[a] Experiment (ft = overexpression of, and complementation of *ft-10* mutant by, *CcFT*, tfl1 = overexpression of, and complementation of the *tfl1-1* mutant by, *CcTFL1A*).

[b] Number of T_1 plants observed for each line.

Supplementary Table S6. Phenotypic distributions in T_1 lines.

				value																																																
**gene**	**genotype**	**construct**	**trait**	**0**	**1**	**2**	**3**	**4**	**5**	**6**	**7**	**8**	**9**	**10**	**11**	**12**	**13**	**14**	**15**	**16**	**17**	**18**	**19**	**20**	**21**	**22**	**23**	**24**	**25**	**26**	**27**	**28**	**29**	**30**	**31**	**32**	**33**	**34**	**35**	**36**	**37**	**38**	**39**	**40**	**42**	**44**	**46**	**54**	**59**	**61**	**65**	**72**
CcFT	*ft-10*	*ft-10*	dtb	.	.	.	.	.	.	.	.	.	.	.	.	.	.	.	.	.	.	.	.	.	.	.	.	.	.	.	1	3	4	6	3	1	.	.	1	.	.	.	.	.	.	.	.	.	.	.	.	.
CcFT	*ft-10*	35S:*CcFT ft-10*	dtb	.	.	.	.	.	.	.	.	.	.	.	.	.	.	.	.	.	.	.	.	.	.	.	.	.	.	3	1	.	6	4	.	1	1	2	.	.	.	.	.	.	.	.	.	.	.	.	.	.
CcFT	*ft-10*	35S:*AtFT ft-10*	dtb	.	.	.	.	.	.	.	.	.	.	5	5	3	2	.	.	1	2	1	.	.	.	.	.	.	.	.	.	.	.	.	.	.	.	.	.	.	.	.	.	.	.	.	.	.	.	.	.	.
CcFT	Col-0	Col-0	dtb	.	.	.	.	.	.	.	.	.	.	.	.	.	.	.	.	1	6	5	8	.	.	.	.	.	.	.	.	.	.	.	.	.	.	.	.	.	.	.	.	.	.	.	.	.	.	.	.	.
CcFT	Col-0	35S:*CcFT* Col-0	dtb	.	.	.	.	.	.	.	.	.	.	.	.	.	.	.	.	2	4	.	2	5	3	3	1	.	.	.	.	.	.	.	.	.	.	.	.	.	.	.	.	.	.	.	.	.	.	.	.	.
CcFT	Col-0	35S:*AtFT* Col-0	dtb	.	.	.	.	.	.	.	.	.	.	3	3	1	1	6	3	.	2	1	.	.	.	.	.	.	.	.	.	.	.	.	.	.	.	.	.	.	.	.	.	.	.	.	.	.	.	.	.	.
CcFT	*ft-10*	*ft-10*	nr_caul	.	.	.	.	.	.	1	3	12	3	.	.	.	.	.	.	.	.	.	.	.	.	.	.	.	.	.	.	.	.	.	.	.	.	.	.	.	.	.	.	.	.	.	.	.	.	.	.	.
CcFT	*ft-10*	35S:*CcFT ft-10*	nr_caul	.	.	.	.	1	6	1	5	4	1	.	.	.	.	.	.	.	.	.	.	.	.	.	.	.	.	.	.	.	.	.	.	.	.	.	.	.	.	.	.	.	.	.	.	.	.	.	.	.
CcFT	*ft-10*	35S:*AtFT ft-10*	nr_caul	1	6	7	4	.	1	.	.	.	.	.	.	.	.	.	.	.	.	.	.	.	.	.	.	.	.	.	.	.	.	.	.	.	.	.	.	.	.	.	.	.	.	.	.	.	.	.	.	.
CcFT	Col-0	Col-0	nr_caul	.	1	6	11	2	.	.	.	.	.	.	.	.	.	.	.	.	.	.	.	.	.	.	.	.	.	.	.	.	.	.	.	.	.	.	.	.	.	.	.	.	.	.	.	.	.	.	.	.
CcFT	Col-0	35S:*CcFT* Col-0	nr_caul	.	1	4	11	4	.	.	.	.	.	.	.	.	.	.	.	.	.	.	.	.	.	.	.	.	.	.	.	.	.	.	.	.	.	.	.	.	.	.	.	.	.	.	.	.	.	.	.	.
CcFT	Col-0	35S:*AtFT* Col-0	nr_caul	.	4	9	4	3	.	.	.	.	.	.	.	.	.	.	.	.	.	.	.	.	.	.	.	.	.	.	.	.	.	.	.	.	.	.	.	.	.	.	.	.	.	.	.	.	.	.	.	.
CcFT	*ft-10*	*ft-10*	nr_leaf	.	.	.	.	.	.	.	.	.	.	.	.	.	.	.	.	.	.	.	.	.	1	.	.	.	.	2	1	6	3	3	2	1	.	.	.	.	.	.	.	.	.	.	.	.	.	.	.	.
CcFT	*ft-10*	35S:*CcFT ft-10*	nr_leaf	.	.	.	.	.	.	.	.	.	.	.	.	.	.	.	.	.	.	.	1	.	2	2	4	.	3	.	1	2	2	.	1	.	.	.	.	.	.	.	.	.	.	.	.	.	.	.	.	.
CcFT	*ft-10*	35S:*AtFT ft-10*	nr_leaf	.	.	2	2	10	2	3	.	.	.	.	.	.	.	.	.	.	.	.	.	.	.	.	.	.	.	.	.	.	.	.	.	.	.	.	.	.	.	.	.	.	.	.	.	.	.	.	.	.
CcFT	Col-0	Col-0	nr_leaf	.	.	.	.	.	.	.	.	3	3	10	3	1	.	.	.	.	.	.	.	.	.	.	.	.	.	.	.	.	.	.	.	.	.	.	.	.	.	.	.	.	.	.	.	.	.	.	.	.
CcFT	Col-0	35S:*CcFT* Col-0	nr_leaf	.	.	.	.	.	.	.	.	1	4	4	3	1	2	4	1	.	.	.	.	.	.	.	.	.	.	.	.	.	.	.	.	.	.	.	.	.	.	.	.	.	.	.	.	.	.	.	.	.
CcFT	Col-0	35S:*AtFT* Col-0	nr_leaf	.	.	2	4	8	4	.	.	1	1	.	.	.	.	.	.	.	.	.	.	.	.	.	.	.	.	.	.	.	.	.	.	.	.	.	.	.	.	.	.	.	.	.	.	.	.	.	.	.
CcFT	*ft-10*	*ft-10*	nr_leaf_total	.	.	.	.	.	.	.	.	.	.	.	.	.	.	.	.	.	.	.	.	.	.	.	.	.	.	.	1	.	.	.	.	.	1	1	2	5	2	4	1	2	.	.	.	.	.	.	.	.
CcFT	*ft-10*	35S:*CcFT ft-10*	nr_leaf_total	.	.	.	.	.	.	.	.	.	.	.	.	.	.	.	.	.	.	.	.	.	.	.	.	1	1	1	2	2	1	1	.	2	1	1	1	2	1	1	.	.	.	.	.	.	.	.	.	.
CcFT	*ft-10*	35S:*AtFT ft-10*	nr_leaf_total	.	.	.	.	3	8	2	1	2	2	1	.	.	.	.	.	.	.	.	.	.	.	.	.	.	.	.	.	.	.	.	.	.	.	.	.	.	.	.	.	.	.	.	.	.	.	.	.	.
CcFT	Col-0	Col-0	nr_leaf_total	.	.	.	.	.	.	.	.	.	1	2	2	2	10	.	3	.	.	.	.	.	.	.	.	.	.	.	.	.	.	.	.	.	.	.	.	.	.	.	.	.	.	.	.	.	.	.	.	.
CcFT	Col-0	35S:*CcFT* Col-0	nr_leaf_total	.	.	.	.	.	.	.	.	.	.	2	2	2	3	3	1	2	.	5	.	.	.	.	.	.	.	.	.	.	.	.	.	.	.	.	.	.	.	.	.	.	.	.	.	.	.	.	.	.
CcFT	Col-0	35S:*AtFT* Col-0	nr_leaf_total	.	.	.	.	3	3	7	3	.	2	1	.	.	1	.	.	.	.	.	.	.	.	.	.	.	.	.	.	.	.	.	.	.	.	.	.	.	.	.	.	.	.	.	.	.	.	.	.	.
CcTFL1	*tfl1-1*	*tfl1-1*	dtb	.	.	.	.	.	.	.	.	.	.	.	.	.	.	.	8	22	7	.	3	.	.	.	.	.	.	.	.	.	.	.	.	.	.	.	.	.	.	.	.	.	.	.	.	.	.	.	.	.
CcTFL1	*tfl1-1*	pAtTFL1:*CcTFL1 tfl1-1*	dtb	.	.	.	.	.	.	.	.	.	.	.	.	.	.	4	3	4	9	2	2	3	1	1	.	.	.	.	.	.	.	.	.	.	.	.	.	.	.	.	.	.	.	.	.	.	.	.	.	.
CcTFL1	*tfl1-1*	35S:*CcTFL1 tfl1-1*	dtb	.	.	.	.	.	.	.	.	.	.	.	.	.	.	.	.	.	.	.	1	.	.	.	1	.	2	.	.	.	.	1	.	3	.	.	.	.	.	.	.	.	.	.	1	.	.	.	.	.
CcTFL1	Col-0	Col-0	dtb	.	.	.	.	.	.	.	.	.	.	.	.	.	.	.	.	1	5	3	12	13	.	3	3	.	.	.	.	.	.	.	.	.	.	.	.	.	.	.	.	.	.	.	.	.	.	.	.	.
CcTFL1	Col-0	35S:*CcTFL1* Col-0	dtb	.	.	.	.	.	.	.	.	.	.	.	.	.	.	.	.	.	.	.	.	1	1	1	.	2	2	.	2	1	2	4	1	1	.	1	3	.	1	1	1	.	.	1	.	.	.	.	.	.
CcTFL1	*tfl1-1*	*tfl1-1*	nr_caul	.	21	19	.	.	.	.	.	.	.	.	.	.	.	.	.	.	.	.	.	.	.	.	.	.	.	.	.	.	.	.	.	.	.	.	.	.	.	.	.	.	.	.	.	.	.	.	.	.
CcTFL1	*tfl1-1*	pAtTFL1:*CcTFL1 tfl1-1*	nr_caul	.	2	23	4	.	.	.	.	.	.	.	.	.	.	.	.	.	.	.	.	.	.	.	.	.	.	.	.	.	.	.	.	.	.	.	.	.	.	.	.	.	.	.	.	.	.	.	.	.
CcTFL1	*tfl1-1*	35S:*CcTFL1 tfl1-1*	nr_caul	1	3	.	1	.	.	.	1	.	.	.	2	.	.	.	.	.	.	.	.	.	.	.	.	.	.	.	.	.	.	1	.	.	.	.	.	.	.	.	.	.	.	.	.	.	.	.	.	.
CcTFL1	Col-0	Col-0	nr_caul	.	1	14	24	1	.	.	.	.	.	.	.	.	.	.	.	.	.	.	.	.	.	.	.	.	.	.	.	.	.	.	.	.	.	.	.	.	.	.	.	.	.	.	.	.	.	.	.	.
CcTFL1	Col-0	35S:*CcTFL1* Col-0	nr_caul	.	.	1	3	2	3	1	3	1	4	.	.	.	1	.	1	1	1	.	.	.	.	.	.	.	.	.	.	.	.	.	.	.	1	.	1	.	.	.	.	.	1	.	1	.	.	.	.	.
CcTFL1	*tfl1-1*	*tfl1-1*	nr_leaf	.	.	.	.	.	.	3	19	16	2	.	.	.	.	.	.	.	.	.	.	.	.	.	.	.	.	.	.	.	.	.	.	.	.	.	.	.	.	.	.	.	.	.	.	.	.	.	.	.
CcTFL1	*tfl1-1*	pAtTFL1:*CcTFL1 tfl1-1*	nr_leaf	.	.	.	.	.	.	.	6	6	13	3	1	.	.	.	.	.	.	.	.	.	.	.	.	.	.	.	.	.	.	.	.	.	.	.	.	.	.	.	.	.	.	.	.	.	.	.	.	.
CcTFL1	*tfl1-1*	35S:*CcTFL1 tfl1-1*	nr_leaf	.	.	.	.	.	1	.	.	.	1	3	.	1	.	1	.	1	.	.	.	.	.	.	.	.	.	.	.	.	.	.	1	.	.	.	.	.	.	.	.	.	.	.	.	.	.	.	.	.
CcTFL1	Col-0	Col-0	nr_leaf	.	.	.	.	.	.	.	3	16	13	7	.	1	.	.	.	.	.	.	.	.	.	.	.	.	.	.	.	.	.	.	.	.	.	.	.	.	.	.	.	.	.	.	.	.	.	.	.	.
CcTFL1	Col-0	35S:*CcTFL1* Col-0	nr_leaf	.	.	.	.	.	.	.	.	.	.	2	2	1	4	4	2	2	1	1	2	1	1	.	1	1	.	1	.	.	.	.	.	.	.	.	.	.	.	.	.	.	.	.	.	.	.	.	.	.
CcTFL1	*tfl1-1*	*tfl1-1*	nr_leaf_total	.	.	.	.	.	.	.	.	13	19	7	1	.	.	.	.	.	.	.	.	.	.	.	.	.	.	.	.	.	.	.	.	.	.	.	.	.	.	.	.	.	.	.	.	.	.	.	.	.
CcTFL1	*tfl1-1*	pAtTFL1:*CcTFL1 tfl1-1*	nr_leaf_total	.	.	.	.	.	.	.	.	.	6	8	9	4	1	1	.	.	.	.	.	.	.	.	.	.	.	.	.	.	.	.	.	.	.	.	.	.	.	.	.	.	.	.	.	.	.	.	.	.
CcTFL1	*tfl1-1*	35S:*CcTFL1 tfl1-1*	nr_leaf_total	.	.	.	.	.	.	.	.	1	1	.	3	.	.	.	.	.	.	.	1	.	.	.	.	.	1	.	1	.	.	.	.	.	.	.	.	.	.	.	.	.	.	.	.	.	.	1	.	.
CcTFL1	Col-0	Col-0	nr_leaf_total	.	.	.	.	.	.	.	.	1	.	9	11	14	4	1	.	.	.	.	.	.	.	.	.	.	.	.	.	.	.	.	.	.	.	.	.	.	.	.	.	.	.	.	.	.	.	.	.	.
CcTFL1	Col-0	35S:*CcTFL1* Col-0	nr_leaf_total	.	.	.	.	.	.	.	.	.	.	.	.	.	3	.	2	.	1	1	2	2	1	2	1	.	2	1	.	.	.	.	1	.	.	1	1	.	1	.	.	.	.	.	.	1	1	.	1	1
